# Glioma chemotherapeutic resistance is tied to membrane electrophysiological properties and glycosylation

**DOI:** 10.1002/btm2.70069

**Published:** 2025-09-22

**Authors:** Alan Y. L. Jiang, Andrew R. Yale, J. Nicole Hanamoto, Nicole S. Lav, Vi Phuong Dang, Clarissa C. Ro, Christopher R. Douglas, Kaijun Di, Jacob Deyell, Daniela A. Bota, Lisa A. Flanagan

**Affiliations:** ^1^ Department of Biomedical Engineering University of California Irvine Irvine California USA; ^2^ Sue & Bill Gross Stem Cell Research Center University of California Irvine Irvine California USA; ^3^ Department of Anatomy & Neurobiology University of California Irvine Irvine California USA; ^4^ Department of Neurology University of California Irvine Irvine California USA; ^5^ Department of Pathology & Laboratory Medicine University of California Irvine Irvine California USA; ^6^ Chao Family Comprehensive Cancer Center University of California Irvine Orange California USA

**Keywords:** cell sorting, chemoresistance, dielectrophoresis, glioblastoma, temozolomide

## Abstract

Diffuse gliomas are brain tumors that include oligodendroglioma, astrocytoma, and glioblastoma (GBM), the most common and deadly primary brain tumor. A major challenge in glioma treatment is resistance to the first‐line chemotherapeutic, temozolomide (TMZ). Plasma membrane properties of cells with increased chemotherapeutic resistance are not well understood, despite the fact that the membrane is the first point of contact with the environment and greatly shapes cell behavior. Plasma membrane glycosylation impacts cell function, and we found significant differences in glycosylation of TMZ‐resistant cells. We further identified plasma membrane electrophysiological properties predicting glioma cell TMZ resistance. We enriched cells with higher TMZ resistance by sorting glioma cells based on electrophysiological properties, indicating the relevance of membrane properties to chemotherapeutic resistance. These findings could lead to rapid separation methods for patient tumor cells, a better understanding of the molecular profiles of resistant cells, and novel treatment options for gliomas.

**Translational Impact Statement**

Brain cancers, such as gliomas, are particularly deadly due to the presence of cells resistant to treatment. Characterizing these resistant glioma cells is critical for understanding why they are resistant and for identifying new therapies. We found resistant glioma cells differ in plasma membrane sugars and electrophysiological properties, and utilized electrophysiological differences to enrich resistant cells. This novel cell enrichment enables further analyses to identify biomarkers and mechanisms of resistance, leading to more effective treatments for glioma.

## 1 INTRODUCTION

Diffuse gliomas infiltrate surrounding nervous system tissue, and tumor types include oligodendroglioma, astrocytoma, and glioblastoma (GBM). GBM is the most prevalent primary brain tumor, with an incidence of approximately 13,000 cases annually in the United States.^1^ Despite surgery and treatment with radiation and chemotherapeutics, median survival is only ~9 months, making GBM particularly deadly.^1^, ^2^

Resistance to temozolomide (TMZ), the first‐line chemotherapy agent for glioma, is a major problem. Patients who initially respond to TMZ often relapse, and further TMZ treatment fails, at which point patient survival is severely limited by a lack of alternative treatments.^3^ TMZ resistance is linked to multiple cellular factors, including the expression of DNA repair enzymes, membrane transporters, autophagy‐related proteins, and activation of the mesenchymal program (for recent review see^4^). Acquisition of *de novo* mutations during cancer therapy may give rise to TMZ resistance; however, inherently resistant cells can also exist within the tumor before treatment.^5^ Multiple lines of data suggest that TMZ‐resistant glioma cells commonly act as cancer stem cells and drive tumor recurrence.^6^, ^7^

TMZ‐resistant cells express plasma membrane components that mediate interactions with the tumor microenvironment; these components may aid resistance and provide a means to detect and enrich resistant cells.^7^ Investigation of resistant cell plasma membrane composition is thus crucial for building a fuller understanding of resistance mechanisms. Cell surface glycosylation, in which glycans are attached to membrane proteins and lipids, is a major plasma membrane modification that regulates membrane protein function, localization at the cell surface, and ligand affinity.^8^ Glycosylation modulates the activity of many cell surface proteins, including growth factor receptors, adhesion proteins, and membrane transporters, which could all have profound functional consequences for glioma drug resistance.

Plasma membrane electrophysiological properties are affected by glycosylation. In neural stem and progenitor cells, amplifying complex cell surface glycans increased the electrophysiological property membrane capacitance and shifted cell fate, indicating the impact of glycosylation on cell function.^9^ A rapid way to assess the plasma membrane electrophysiological properties of large quantities of live cells is through dielectrophoresis (DEP). DEP is a technique in which non‐homogeneous electric fields induce cell movement based on intrinsic cellular properties, obviating the need for cell type‐specific labels.^11^ DEP can be used to measure cell membrane and cytoplasm electrophysiological properties and separate cells based on their intrinsic electrophysiological differences.^12^, ^13^, ^14^, ^15^ Importantly, DEP is not toxic for most cell types in the short time frames needed for cell analysis and sorting,^16^ so live cells can be enriched for downstream analyses.

DEP is useful for detecting and separating cells with subtle phenotypic differences.^13^, ^15^, ^17^ For example, neural stem and progenitor cells that differ in fate bias can be selectively enriched using DEP since they differ in whole‐cell membrane capacitance, which reflects the membrane's ability to store charge.^14^, ^18^, ^19^, ^20^, ^21^, ^22^, ^23^ Membrane capacitance is a robust marker of cell phenotype as it distinguishes cells with different fates in multiple stem cell lineages (neural, hematopoietic, mesenchymal/adipose‐derived, and embryonic).^15^

Discovering new approaches for rapidly identifying and isolating TMZ‐resistant cells from resected patient tumors will improve understanding of their molecular characteristics, resistance mechanisms, and potential treatment strategies.^24^ Enriching resistant cells commonly involves extensive tumor cell culture in TMZ to select for resistant cells. However, this is a lengthy process (weeks to months), and prolonged TMZ exposure could induce cellular changes not directly correlated with resistance or reflective of resistant cell phenotypes in the native tumor microenvironment.^25^ Thus, there is a need for alternative strategies to rapidly enrich TMZ‐resistant cells and bypass extended culture in TMZ.

To explore plasma membrane contributions to TMZ resistance, we tested whether TMZ‐resistant glioma cells possess distinct glycosylation patterns and whether they could be identified and enriched by their electrophysiological properties.

## 2 RESULTS

### 2.1 Cell surface N‐glycans differ between TMZ‐resistant and control glioma cells

We found previously that neural stem and progenitor cell differentiation and fate are linked to cell surface N‐glycosylation.^9^, ^10^ Since glycosylation regulates multiple plasma membrane proteins that could impact resistance, we assessed glycosylation of glioma cells differing in TMZ resistance. We utilized D54 and U251 GBM cells cultured in TMZ to select for resistant cells (D54‐TR, U251‐TR) and compared them to controls cultured without TMZ, either in regular media or in media with DMSO (diluent for TMZ). Extended growth in TMZ greatly increased the TMZ resistance of D54 and U251 cells (Figure S1).

**FIGURE 1:**
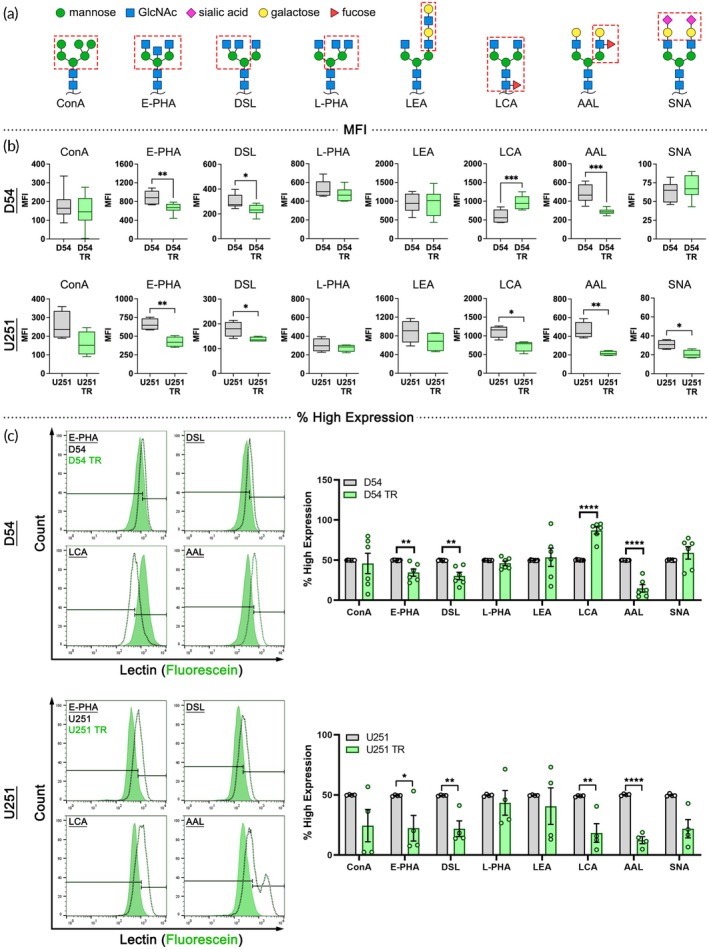
TMZ‐resistant cells differ in cell surface glycosylation. (a) Schematics of N‐glycan structures; symbols denote particular sugar moieties. Boxed regions indicate structures recognized by lectins: *Canavalia ensiformis* concanavalin A (ConA), *Phaseolus vulgaris* erythroagglutinin (E‐PHA), *Datura stramonium* lectin (DSL), *Phaseolus vulgaris* leucoagglutinin (L‐PHA), *Lycopersicon esculentum* agglutinin (LEA), *Lens culinaris* agglutinin (LCA), *Aleuria aurantia* lectin (AAL), *Sambucus nigra* agglutinin (SNA). (b) Analysis of mean fluorescence intensity (MFI) showed that D54‐TR and U251‐TR cells had reduced levels of E‐PHA, DSL, and AAL binding. Relative to controls, D54‐TR cells had higher levels of LCA binding, but U251‐TR cells had lower levels. U251‐TR cells had lower SNA binding compared to controls. (c) Histogram plots show peaks for TR cells and controls and the 50% gating of controls into subpopulations with low and high expression. Bar graphs show the percentage of TR cells with high lectin expression relative to controls (set to 50%) and reveal significant differences in E‐PHA, DSL, LCA, and AAL for both D54 and U251 control cells compared to TR cells. Box plots in b show median with min to max whiskers and bars in c, show SEM, *n* ≥ 3, unpaired Student's *t*‐test control versus TR, *p < 0.05, **p < 0.01, ***p < 0.001, ****p < 0.0001.

To assess the plasma membrane glycome of TMZ‐resistant and control cells, we used lectin flow cytometry to detect common cell surface N‐glycans: ConA for high mannose, E‐PHA for bisecting, DSL for β1–4 branched, L‐PHA for β1–6 branched, LEA for N‐acetyllactosamine, LCA for core fucose, AAL for outer fucose, and SNA for terminal sialic acid (Figure 1a). Analysis of lectin mean fluorescence intensity (MFI) revealed significantly decreased binding of E‐PHA, DSL, and AAL to TMZ‐resistant cells compared to controls for both D54 and U251, showing consistent changes in glycosylation associated with increased resistance across two GBM cell types (Figure 1b). LCA lectin binding was significantly higher on D54‐TR cells compared to controls, but the pattern was opposite for U251 cells (Figure 1b). SNA lectin binding was significantly lower on U251‐TR cells compared to controls, while D54‐TR cells had no difference compared to controls (Figure 1b). Thus, core fucose (detected by LCA) and sialic acid (detected by SNA) were not consistently tied to TMZ resistance across D54 and U251 cells.

MFI provides a measure of the entire cell population. To measure the proportion of cells with high or low levels of lectin binding, we used control D54 and U251 histograms to gate cells into low and high expressing subpopulations. We quantified the percentage of TR cells in the high expressing gate and found patterns similar to those identified with MFI analysis. The high expressing population for E‐PHA, DSL, and AAL was reduced in D54‐TR cells compared to D54, but was increased for LCA (Figure 1c). U251‐TR cells displayed similar trends for E‐PHA, DSL, and AAL, but had a reduction of the LCA high expressing population (Figure 1c). In summary, both D54‐TR and U251‐TR cells consistently exhibited decreased binding of E‐PHA, DSL, and AAL lectins compared to controls, indicating changes in bisecting and branched N‐glycans and outer fucose containing N‐glycans associated with TMZ resistance.

### 2.2 TMZ‐resistant glioma cells have distinct electrophysiological properties

**FIGURE 2:**
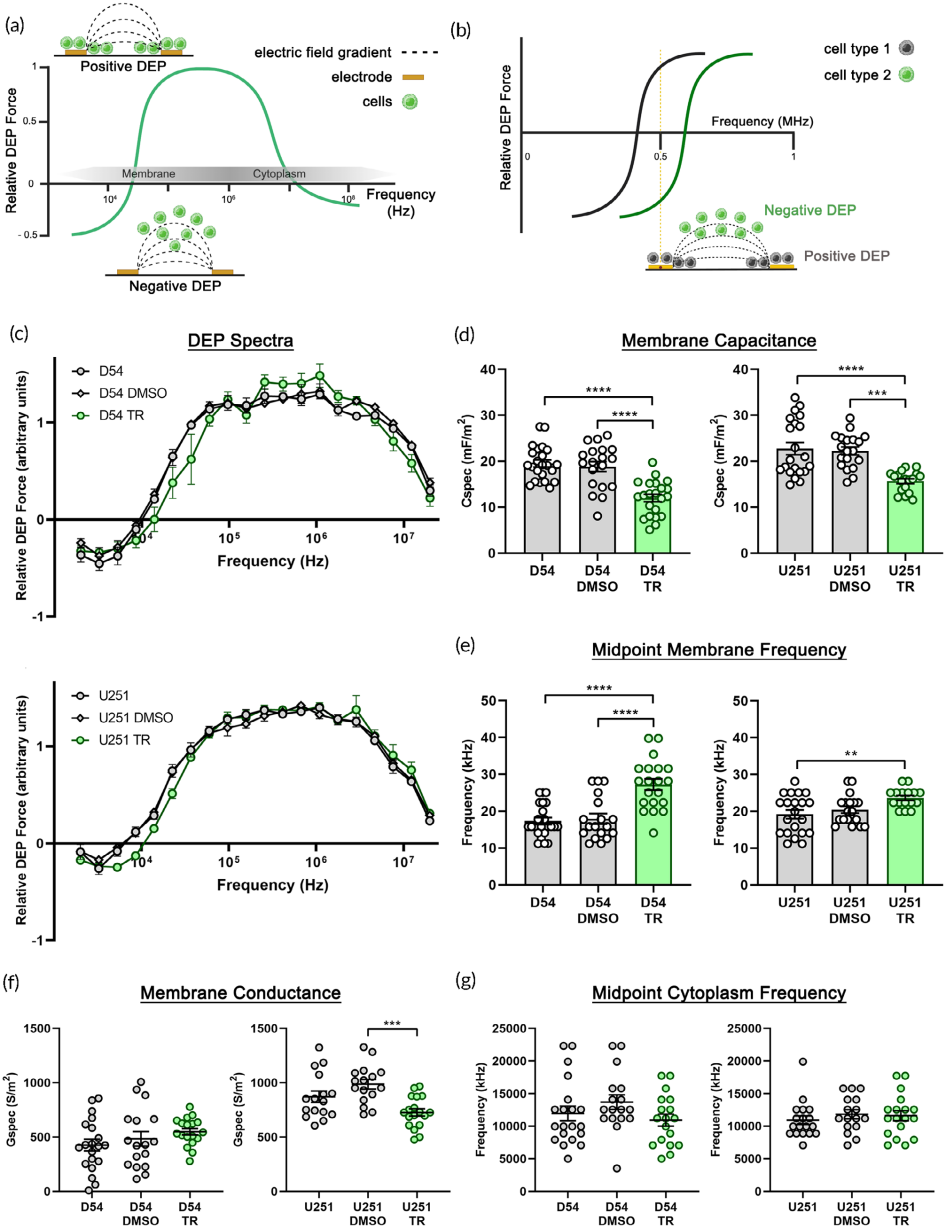
TMZ‐resistant cells and controls differ in membrane electrophysiological properties. (a) Schematic showing cell behavior in DEP. The behavior of a cell population in DEP is graphed as the magnitude of the relative DEP force, which is responsible for cell movement, across the applied electric field frequency range. Lower frequencies are dominated by cell plasma membrane properties while higher frequencies probe the cytoplasm. Insets show electrodes, with electric field lines (dashed lines) indicating the electric field gradient, and cells in positive DEP and negative DEP. (b) Schematic showing the behavior of two cell types in DEP that have different DEP responses. At an applied frequency of 0.5 MHz, the gray cells experience positive DEP and collect along electrode edges at the high strength part of the electric field, while the green cells experience negative DEP and are repelled away from the high strength electric field region. (c) DEP spectra of D54 (D54, D54‐DMSO, D54‐TR) and U251 (U251, U251‐DMSO, U251‐TR) cells show the relative DEP force across applied frequencies. For both sets of cells, DEP spectra of TMZ‐resistant (TR) cells are shifted rightward compared to controls in the lower frequency range, which reflects plasma membrane properties. (d) Specific membrane capacitance (Cspec) values for TR cells were lower than those of controls. (e) Midpoint membrane frequency of TR cells was higher than those of controls. (f) There was no significant difference in the specific membrane conductance (Gspec) values of D54 controls and TR cells, but U251‐TR cells had lower values than U251‐DMSO controls. (g) The midpoint cytoplasm frequency of the cells did not significantly differ. Error bars show SEM, *n* ≥ 3, one‐way ANOVA, Tukey *post hoc* for multiple comparisons, **p < 0.01, ***p < 0.001, ****p < 0.0001.

We found previously that neural stem and progenitor cells differing in cell surface N‐glycosylation also differ in whole‐cell electrophysiological properties.^9^ We measured the whole‐cell electrophysiological properties of control and TMZ‐resistant cells using alternating current DEP across a frequency range.^9^, ^11^, ^19^, ^23^, ^26^ Cells experience a frequency‐dependent induced DEP force that causes either repulsion from the high strength region of the electric field, which is termed negative DEP, or attraction to the high strength region (positive DEP) (Figure 2a).

Movement of cells in DEP at lower frequencies is dominated by plasma membrane properties, while cytoplasmic properties dominate at higher frequencies (Figure 2a).^12^ If two cells respond to different frequencies in DEP due to their inherent cellular properties, a frequency can be chosen at which one cell type will experience positive DEP and the other negative DEP (Figure 2b). This can be used to measure distinct cell electrophysiological properties and to sort cells in DEP‐based devices.

DEP spectra reflect cell movement across different frequencies of the applied electric field. We measured the DEP spectra of control and TMZ‐resistant cells using a 3DEP analyzer (see Section 4) and found the spectra of resistant cells (D54‐TR and U251‐TR) were distinct from those of control cells (D54 and U251) (Figure 2c). Multiple whole‐cell electrophysiological properties can be extracted from DEP spectra, including membrane capacitance (ability to store charge), membrane conductance (ability to transmit charge), and midpoint frequencies (cell type‐specific frequency responses).^26^ We found whole‐cell membrane capacitance values of TMZ‐resistant cells were significantly lower than those of controls (Figure 2d). Midpoint membrane frequency, which is dominated by the plasma membrane, was significantly higher for TMZ‐resistant cells than controls (Figure 2e). Whole‐cell membrane conductance values were not consistently different between TMZ‐resistant and control cells (Figure 2f). Similarly, the midpoint cytoplasm frequencies of TMZ‐resistant and control cells did not differ (Figure 2g).

### 2.3 Parameter optimization for DEP‐based sorting of glioma cells

For DEP‐based sorting, cells must maintain high viability after electric field exposure at sorting frequencies and voltages.^16^ DEP sorting in our device (described below) occurs in <1 min, so we tested D54 cell viability after 1 or 5 min in electric fields to be conservative. D54 cells maintained >80% viability for all conditions tested except the highest voltage (10 V) and longest time exposure (5 min) (Figure S2). These data established voltage and time limits for sorting.

Cell sorting in alternating current DEP requires cell suspension in an osmotically balanced, low conductivity buffer.^11^, ^18^ We tested the viability of D54 cells in the DEP buffer we used for mouse and human neural stem and progenitor cell sorting.^16^, ^18^, ^19^, ^22^, ^23^ We incubated cells in DEP buffer for up to 6 h (the maximum time in buffer during sorting) and tested whether viability was affected by incubation temperature (ice or room temperature). All samples showed high viability (>80%) after incubation, showing that DEP buffer did not acutely impact cell viability (Figure S3a–c).

After the 6‐h buffer incubation, we plated cells in normal growth conditions and assessed continued survival and growth using phase contrast imaging 1–2 days later. D54 cells incubated in DEP buffer did not recover well; images showed reduced cell numbers and higher cell death compared to controls (Figure S3d). This was surprising since we did not observe reduced survival of neural stem and progenitor cells with this buffer.^16^, ^18^, ^19^, ^22^, ^23^ By testing the duration of buffer incubation, we found lower D54 cell number and greater cell death after 2 h in DEP buffer (Figure S3e).

**FIGURE 3:**
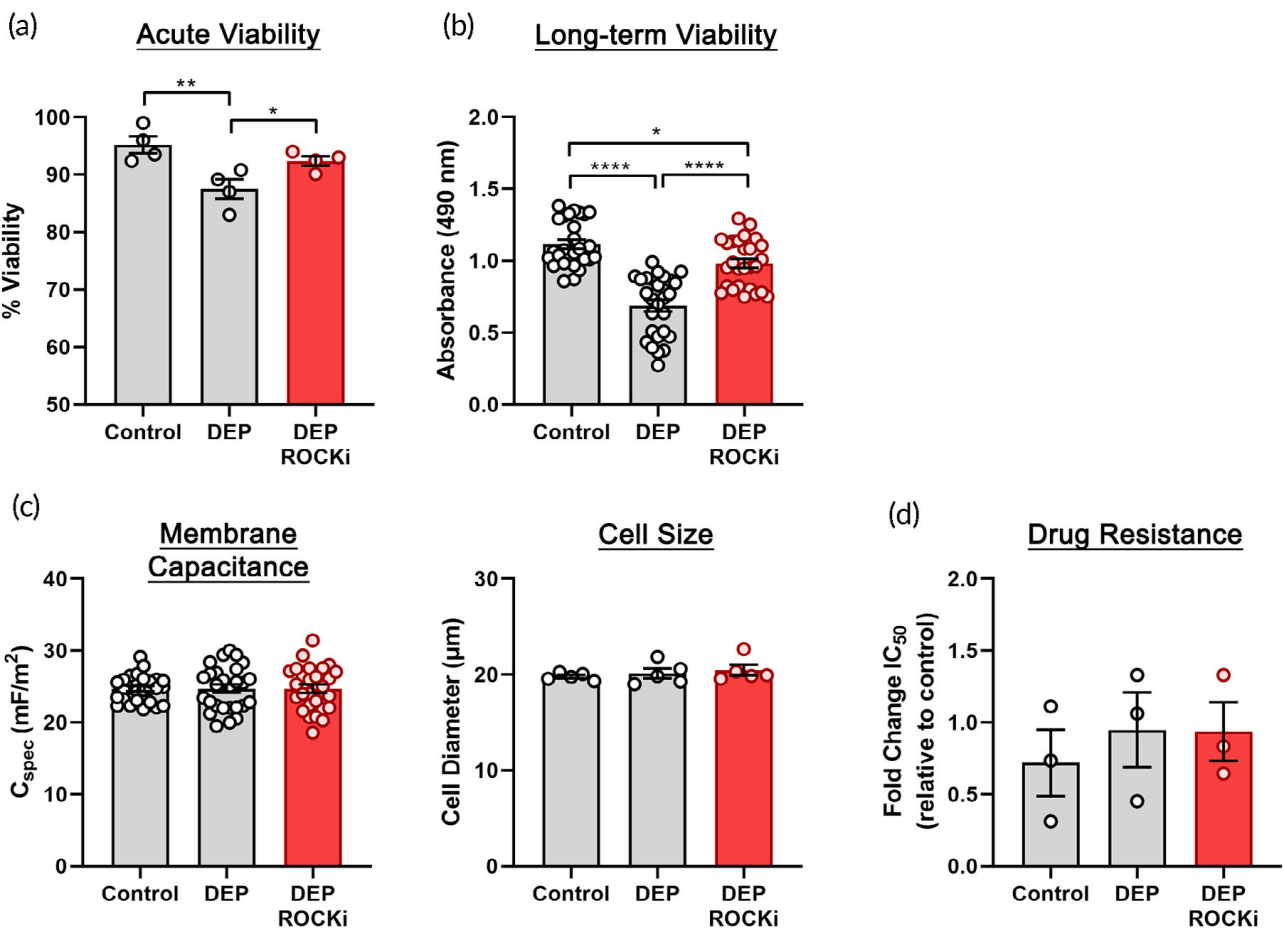
Optimization of buffer conditions for DEP‐based sorting of GBM cells. (a) Viability of D54 cells immediately after 6 h room temperature incubation in regular growth media (control), DEP buffer, or DEP buffer supplemented with 5 μM ROCKi was measured by trypan blue staining. Addition of ROCKi improved viability in the DEP buffer. (b) After 6 h incubation in media or buffers, D54 cells were plated in normal growth conditions and the number of viable cells measured after 2 days by XTT assay. DEP buffer with 5 μM ROCKi significantly increased the number of viable cells compared to DEP buffer alone. (c) Incubation of D54 cells for 6 h in DEP buffer or DEP buffer with 5 μM ROCKi did not change membrane capacitance or cell diameter. Acutely isolated cells in media served as control. (d) D54 cells acutely isolated in media or incubated in media (control), DEP buffer, or DEP buffer with ROCKi for 6 hours were plated and allowed to recover for 1 day before treatment with TMZ to assess resistance. TMZ IC_50_ is shown as fold change relative to cells acutely isolated in media, and there was no difference in TMZ resistance across samples. Error bars show SEM, *n* ≥ 3, one‐way ANOVA, Tukey *post hoc* for multiple comparisons, *p < 0.05, **p < 0.01, ***p < 0.001, ****p < 0.0001.

We noted that cells incubated in media at room temperature for 6 h had the best survival and growth (Figure S3d) and images showed cell clusters, suggesting that cell–cell contact may improve survival (Figure S3c). Previous studies of human embryonic stem cells that require cell–cell contact for survival found the inclusion of ROCK inhibitor (ROCKi) improved dissociated cell viability.^27^, ^28^ We added ROCKi (1–10 μM) to the DEP buffer and found a concentration of 5 μM ROCKi maintained high D54 survival (Figure S4a,b). ROCKi addition to DEP buffer significantly improved D54 acute (Figure 3a) as well as long‐term (Figure 3b) viability. We tested an alternative RBC‐DEP buffer that contained calcium and magnesium,^29^ but this buffer (with or without 5 μM ROCKi) did not increase D54 viability compared to DEP buffer with 5 μM ROCKi (Figure S4b).

We tested whether ROCKi altered cell parameters that would affect DEP‐based sorting. We measured membrane capacitance and cell size since these directly impact cell responses to the electric field. Addition of 5 μM ROCKi did not change D54 membrane capacitance values or size (Figures 3c, S4c,d). Since we aimed to isolate TMZ‐resistant cells, we tested whether ROCKi affected D54 TMZ sensitivity. To mimic cell sorting conditions, cells were incubated in DEP buffer with ROCKi for 6 h and subsequently plated in normal growth medium without ROCKi. ROCKi in the DEP buffer did not alter TMZ resistance (Figures 3d, S4e). Thus, DEP buffer supplemented with ROCKi improves D54 cell health without altering other parameters that would confound sorting results.

### 2.4 TMZ‐resistant glioma cells can be sorted by electrophysiological properties

**FIGURE 4:**
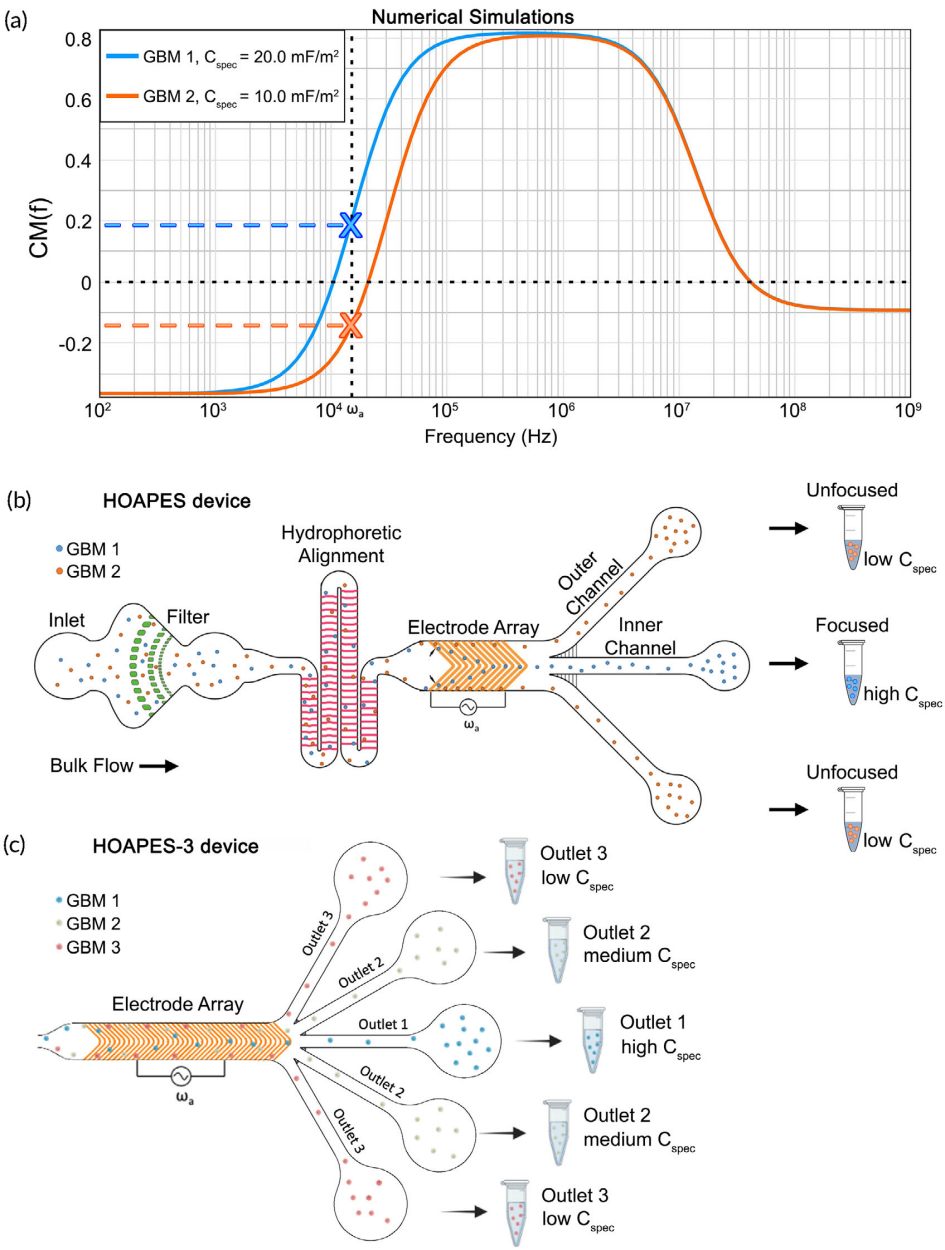
Modeling indicates potential for DEP‐based sorting of TMZ‐resistant cells using HOAPES and HOAPES‐3 devices. (a) Using a single shell model, the Clausius‐Mossotti factor, CM(f), was modeled as a function of frequency of the applied electric field for hypothetical cells with membrane capacitance (Cspec) of either 20 mF/m^2^ (GBM1, similar to control D54 cells, blue line) or 10 mF/m^2^ (GBM2, similar to D54‐TR cells, orange line) (see Figure 1b). Media conductivity was set to 100 μS/cm. In the low frequency dispersion region, an applied frequency (ω_a_) can be identified at which GBM1 cells have a positive CM(f), indicated by the blue dashed line, while the CM(f) for GBM2 cells is negative (orange dashed line). At this frequency, cells would differ in induced DEP force and be separated: GBM1 cells (control) experience positive DEP and GBM2 cells (TR) experience negative DEP. (b) Schematic of GBM1 (blue) and GBM2 (orange) cells separated in the HOAPES device using an applied frequency (ω_a_). GBM1 cells experience positive DEP, focusing along electrodes to the inner channel, while GBM2 cells in negative DEP remain unfocused along the channel edges and exit the outer channels. Focused cells are expected to have higher membrane capacitance than unfocused cells. (c) Schematic depicting HOAPES‐3 device DEP separation region (electrode array) and multiple outlets generating three sorted fractions of low, medium and high membrane capacitance.

TMZ‐resistant cells and controls significantly differed in membrane capacitance and midpoint membrane frequency, suggesting glioma cells could be sorted with DEP to enrich highly resistant cells (Figure 2). To identify ideal sorting parameters, we modeled the behavior of two hypothetical cells with different average membrane capacitance values of 10 and 20 mF/m^2^, as indicated by our D54 and D54‐TR data (Figure 2). The Clausius‐Mossotti (CM) factor is a function of the electrical properties of the cell and its surrounding medium and can predict the behavior of cells in DEP at distinct applied frequencies of the electric field. Graphs of the CM factor across electric field frequency indicated frequencies at which the cells with 20 mF/m^2^ membrane capacitance would experience positive DEP (CM factor above 0) while the 10 mF/m^2^ capacitance cells would undergo negative DEP (CM factor below 0) (Figure 4a). This modeling suggests that glioma cells could be separated in DEP based on their TMZ resistance.

To sort glioma cells, we used DEP‐based cell separation devices designed to increase sorted cell throughput.^22^, ^23^ The devices include a microfluidic channel with three functional regions: PDMS posts to filter out cell clumps, a hydrophoretic alignment section to direct cells into two separate streams along the channel walls, and an electrode array for DEP sorting (Figure 4b).^22^, ^23^ Movies associated with our previous publication show cells traversing the filter and hydrophoretic alignment sections.^22^ After the hydrophoretic alignment section directs cells to the channel edges, cells enter the DEP sorting region (Movie S1, *t* = 0 s). The electrodes for DEP separation are arranged in a chevron pattern such that cells experiencing positive DEP are focused to the center of the channel due to the induced DEP force transporting cells along electrode edges and the fluid hydrodynamic force moving cells down the channel (Movie S1).^22^ Increased spacing between electrodes at the chevron tips allows cells to release and move down the channel from one electrode to another until cells exit via the central outlet (Movie S1, *t* = 6 s). Cells experiencing negative DEP do not track along the electrodes and instead remain along the channel walls due to fluid flow and exit the outer channels (Movie S1, *t* = 14 s). When there is no applied electric field, all the cells remain unfocused and exit the outer channels (Movie S1, *t* = 16 s).

We used two versions of our sorting devices, the hydrodynamic oblique angle parallel electrode sorter (HOAPES)^22^ and a modified HOAPES‐3. The HOAPES device has three outlets and generates two fractions: cells collected from the inner channel (cells experiencing positive DEP) are in the focused fraction, while cells pooled from the two outer channels (cells experiencing negative DEP) are in the unfocused fraction (Figure 4b). Cell sorting in microfluidic DEP devices is primarily driven by two factors: cell electrophysiological properties and cell size. If sorted cells are the same size, separation is largely due to electrophysiological properties, such as membrane capacitance that is depicted in the schematic (Figure 4b). However, if sorted cells differ in size, separation can relate to the size difference alone or a combination of size and electrophysiological properties. Thus, it is important to measure the electrophysiological properties and size of sorted cells to determine the primary factors driving sorting.

We developed a new HOAPES‐3 device that has a modified and extended electrode array and 5 outlets to generate three total fractions (Figure 4c). Outlet 1 collects cells experiencing strong positive DEP, outlet 2 weaker positive DEP, and outlet 3 no net force or negative DEP (Figure 4c). The HOAPES‐3 device also incorporates a cell mixing chamber that prevents cell settling during device loading (Figure S5). Both HOAPES and HOAPES‐3 devices sort cells based on individual cell movement, which is due to a combination of the induced DEP force and the fluidic force.^22^

Based on the overlap between D54 and D54‐TR cells in membrane capacitance (D54 range 14.2–27.5 mF/m^2^ and D54‐TR 5.1–19.8 mF/m^2^) (Figure 2), we hypothesized that there could be lower capacitance cells in the D54 population that were more resistant to TMZ. We therefore tested whether DEP sorting of D54 cells would enrich cell populations that differ in TMZ resistance. We sorted D54 cells in the HOAPES and HOAPES‐3 devices, collected sorted cell fractions, measured their electrophysiological properties with 3DEP, and assessed TMZ resistance. TMZ resistance was evaluated by culturing equal numbers of cells in increasing concentrations of TMZ, measuring surviving cell number, and calculating IC_50_ values. Plating defined numbers of cells and plotting absorbance as a function of cell number confirmed the accuracy of surviving cell number determinations in this assay (Figure S6a). We optimized several parameters for the D54 TMZ assays, including cell density, media volume per well, and TMZ treatment duration (Figure S6b).^30^

**FIGURE 5:**
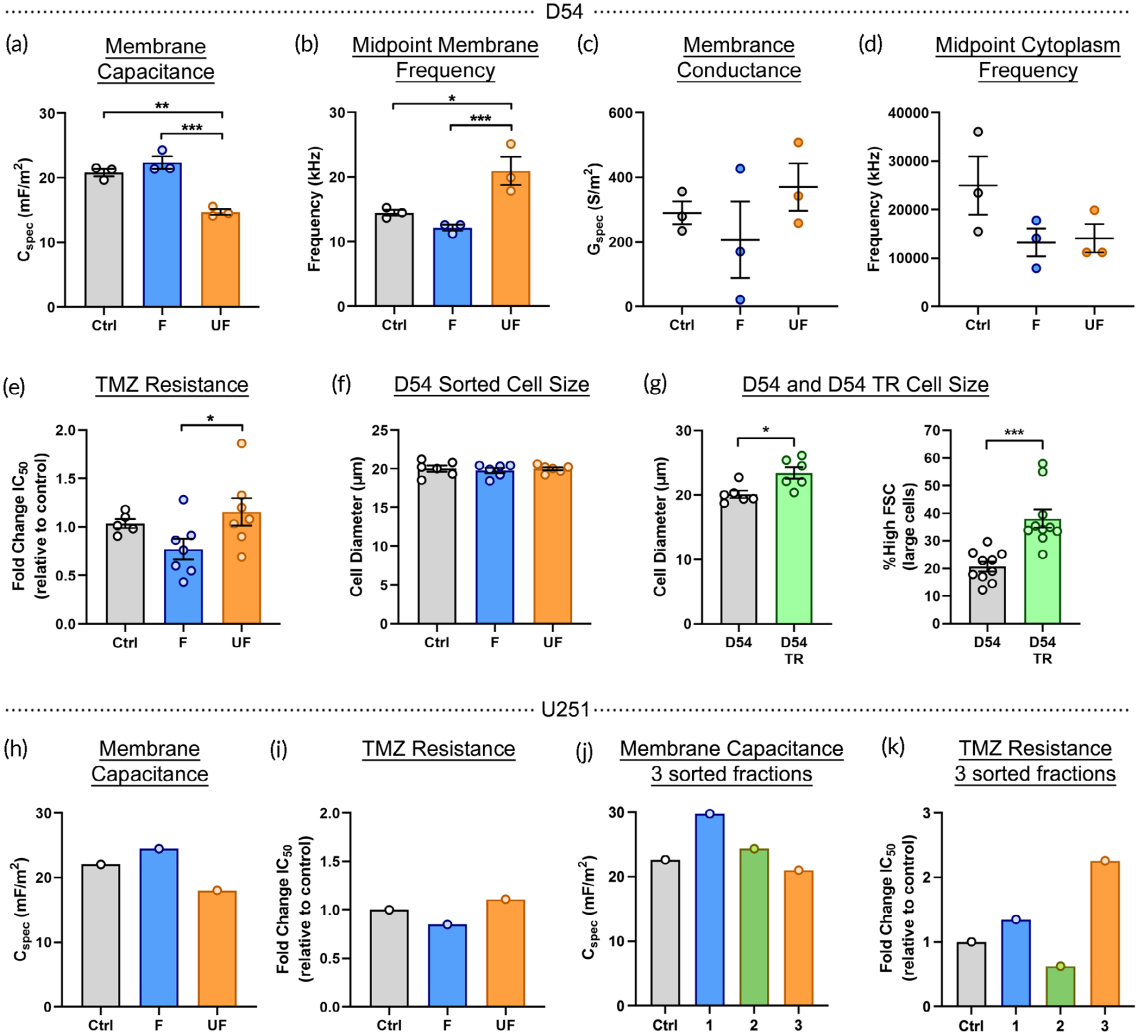
Isolation of TMZ‐resistant cells by membrane electrophysiological properties. (a) D54 GBM cells were sorted into focused (F) and unfocused (UF) fractions using the HOAPES device and immediately analyzed. Cells in the unfocused fraction had lower membrane capacitance values than cells in the focused fraction or controls in DEP buffer. (b) D54 cells in the unfocused fraction had significantly higher midpoint membrane frequency values compared to other samples. (c) D54 cells in the unfocused fraction showed non‐significant increases in membrane conductance compared to cells in the focused fraction. (d) D54 cells in the focused and unfocused fractions did not differ in midpoint cytoplasmic frequency. (e) Fold change in TMZ resistance (IC_50_ values) relative to unsorted controls. Sorted D54 cells were passaged 2–3 times after sorting to generate cells for TMZ assays. Cells in the unfocused fraction were more resistant to TMZ than those in the focused fraction. (f) Sorted D54 cells were analyzed by phase contrast microscopy and focused, unfocused, and unsorted control cells did not differ in size. (g) Analysis of D54 and D54‐TR cell phase contrast images and forward scatter profiles in flow cytometry showed that TR cells were significantly larger than controls (unpaired Student's *t*‐test control vs. TR). (h) Sorted U251 GBM cells showed lower membrane capacitance values for unfocused cells compared to focused cells (*n* = 1). (i) U251 cells in the unfocused fraction were more resistant to TMZ than those in the focused fraction (*n* = 1). (j) U251 cells were sorted in the HOAPES‐3 device and membrane capacitance was measured at three passages post‐sorting. Cells with the highest membrane capacitance were in outlet 1 and the lowest in outlet 3 (*n* = 1). (k) Fold change in TMZ resistance relative to controls was highest for U251 cells sorted to outlet 3 (*n* = 1). Error bars show SEM, *n* ≥ 3 (unless otherwise noted), analyses one‐way ANOVA, Tukey *post hoc* for multiple comparisons (unless otherwise noted), *p < 0.05, **p < 0.01, ***p < 0.001.

D54 cells sorted in the HOAPES device showed significant differences in the electrophysiological properties of unfocused and focused cells. Cells in the unfocused fraction had significantly lower membrane capacitance values than those of cells in the focused fraction (Figure 5a). Cells in the unfocused fraction also had significantly higher midpoint membrane frequency values compared to cells in the focused fraction (Figure 5b). There was no significant difference in the membrane conductance or midpoint cytoplasm frequency of sorted cells (Figure 5c,d). TMZ resistance was significantly higher for cells in the unfocused fraction compared to those in the focused fraction (Figures 5e and S7a). D54 cells sorted with the HOAPES‐3 device produced a similar pattern in that cells with the highest capacitance were collected in outlet 1, while cells with lower capacitance and higher TMZ resistance were in outlet 3 (Figure S7b,c).

Comparison of enriched TMZ‐resistant cells to those made resistant by long term TMZ culture can identify which cellular properties are directly linked to resistance and which are by‐products of continued growth in a chemotherapeutic. For example, sorted D54 cells differ in TMZ resistance (Figure 5e), but do not differ in cell size (Figure 5f). In contrast, D54‐TR cells generated by continuous culture in TMZ were significantly larger than control D54 cells, suggesting larger cell size relates to culture in TMZ (Figure 5g). Sorted cells are advantageous since they can provide insight into resistance mechanisms while avoiding confounding effects of continued TMZ exposure.

We also sorted U251 GBM cells to determine whether sorting was reproducible across different cell lines. Similar to D54 cells, U251 cells in the unfocused fraction had lower membrane capacitance and higher TMZ resistance than focused cells (Figure 5h,i). When U251 cells were sorted with the HOAPES‐3 device, outlet 3 contained cells with lower membrane capacitance and higher TMZ resistance compared to cells in other outlets (Figure 5j,k). As seen with D54 cells, there was no clear trend in membrane conductance and midpoint cytoplasmic frequency of sorted U251 cells (Figure S7d–g). In terms of cell size, sorted U251 cells did not differ in size, but U251‐TR cells grown in TMZ were larger than control U251 cells (Figure S7h), mirroring the pattern observed with D54 cells (Figure 5f,g).

Collectively, these data show that cells with higher TMZ resistance can be enriched using DEP‐based sorting. DEP sorting of glioma cells can help identify properties important for TMZ resistance that are not merely induced by long‐term growth in TMZ.

### 2.5 TMZ‐resistant cells enriched from oligodendroglioma/astrocytoma

Since there are multiple types of diffuse gliomas, we tested whether cells that differ in TMZ resistance could be sorted from other glioma types in addition to GBM. We utilized a human glioma cell line that was originally described as oligodendroglioma, but based on new diagnostic criteria would now be classified as malignant astrocytoma since the cells are IDH wild‐type and do not have the 1p/19q co‐deletion.^2^, ^31^, ^32^ We cultivated these cells in serum‐free conditions as used for patient‐derived human glioma cells and refer to them as HOG‐A to reflect the original name (HOG) and the updated classification as malignant astrocytoma (A). We tested HOG‐A cells in DEP sorting buffer and found reduced viability, which was resolved by the addition of ROCKi without deleterious effects on TMZ resistance (Figure S8a–c). Optimization of TMZ assays determined 3‐day TMZ incubation for HOG‐A cells (Figure S8d).

**FIGURE 6:**
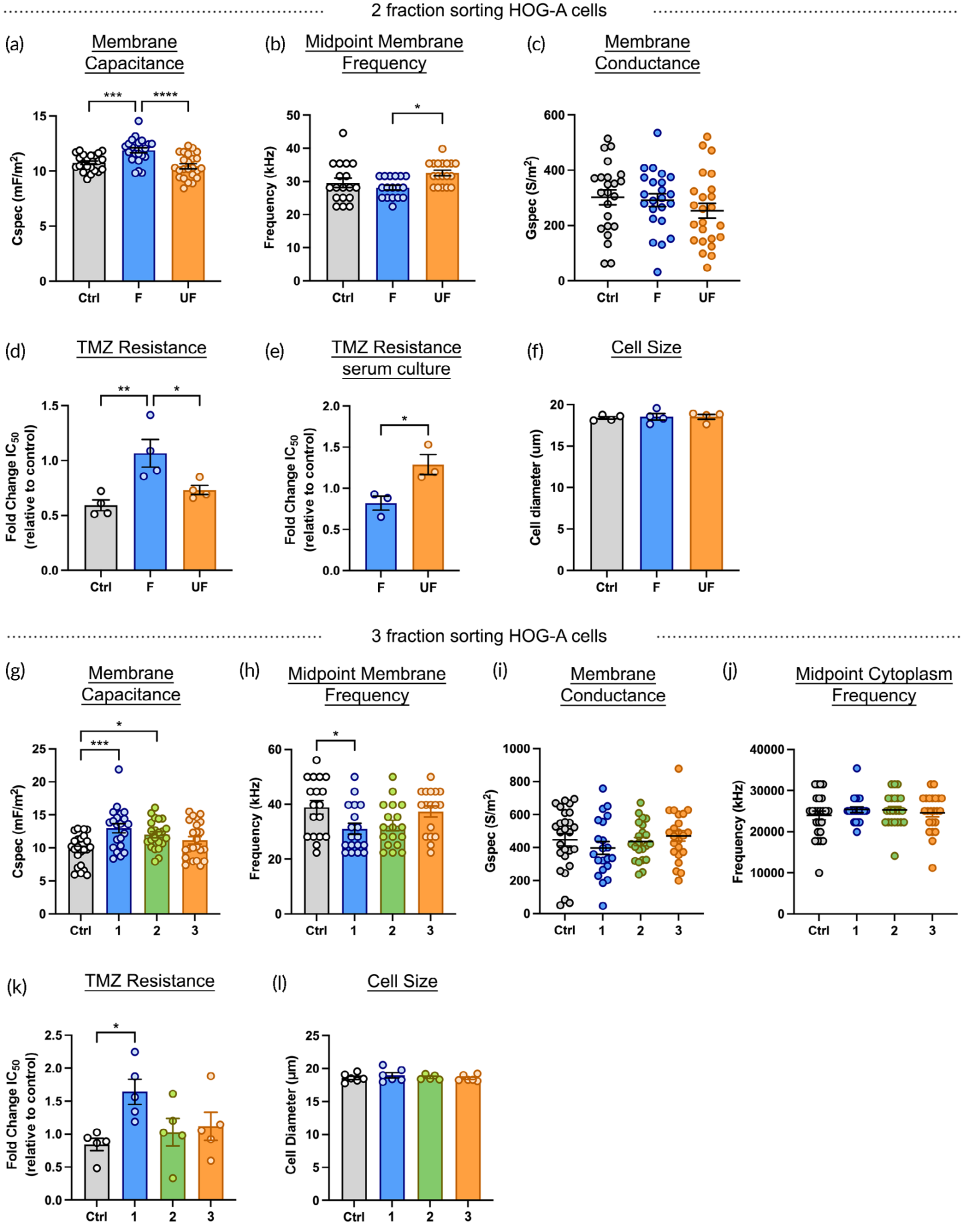
Isolation of TMZ‐resistant cells from a malignant astrocytoma cell line. (a) HOG‐A cells were sorted into focused and unfocused fractions. Cells in the focused fraction had higher membrane capacitance values than cells in the unfocused fraction or controls (cells in DEP buffer and exposed to electric fields). (b) Cells in the unfocused fraction had significantly higher midpoint membrane frequency values compared to cells in the focused fraction. (c) There was no significant difference in membrane conductance among the samples. (d) HOG‐A cells in the focused fraction were more resistant to TMZ than those in the unfocused fraction or media control. Fold change in TMZ resistance (IC_50_ values) is relative to unsorted controls. (e) HOG‐A cells grown in serum prior to DEP sorting showed cells in the unfocused fraction were more resistant to TMZ. (f) Unsorted control and sorted HOG‐A cells did not differ in cell size. (g) HOG‐A cells were sorted into three fractions in the HOAPES‐3 device. Cells collected from outlets 1 and 2 had higher membrane capacitance values than controls in DEP buffer. (h) Sorted HOG‐A cells in outlet 1 had lower midpoint membrane frequency compared to control cells in DEP buffer. (i) There was no significant difference in membrane conductance across sorted cells and controls. (j) The midpoint cytoplasm frequency of sorted HOG‐A cells and controls did not differ. (k) Sorted cells in outlet 1 were more resistant to TMZ compared to those in outlets 2 and 3 or controls in DEP buffer. Fold change in TMZ resistance (IC_50_ values) is relative to unsorted controls. (i) Cell size did not differ among sorted HOG‐A cells or controls. Error bars show SEM, *n* ≥ 3, all analyses one‐way ANOVA, Tukey *post hoc* for multiple comparisons, *p < 0.05, **p < 0.01, ***p < 0.001.

We sorted HOG‐A cells in the HOAPES and HOAPES‐3 devices. As expected, HOG‐A cells showed a frequency‐dependent response to DEP sorting (Movie S2). In the HOAPES device, HOG‐A cells with higher membrane capacitance and lower midpoint membrane frequency were collected in the focused fraction and those with lower capacitance and higher midpoint membrane frequency in the unfocused fraction (Figure 6a,b). All control HOG‐A cells for DEP sorting showed no differences in membrane capacitance or midpoint membrane frequency (Figure S8e,f). As seen with D54 cells, there was no significant difference in membrane conductance of sorted cells in the focused and unfocused fractions (Figure 6c).

HOG‐A cells with the highest TMZ resistance were enriched in the focused fraction, showing that TMZ‐resistant cells can be enriched from other glioma types as well as GBM (Figure 6d). Surprisingly, the pattern of TMZ‐resistant HOG‐A cell enrichment was opposite compared to GBM cells, where higher resistant cells were collected in the unfocused fraction. We hypothesized this may relate to the growth conditions used for the two sets of cells, since D54 and U251 GBM cells were grown in serum and HOG‐A cells were in serum‐free media. To test this, we grew HOG‐A cells in media with and without serum. After 4 passages in serum‐containing media, the HOG‐A DEP spectra shifted, with cells having decreased membrane capacitance and increased midpoint membrane frequency (Figure S8g,h). To test whether these shifts might explain differences in the sorting patterns, we sorted serum‐cultured HOG‐A cells in the HOAPES device. For HOG‐A cells grown in serum, higher TMZ resistance cells were collected in the unfocused fraction, showing that cells differing in TMZ resistance can be enriched by DEP‐based sorting independent of growth conditions (Figure 6e). As seen with D54 and U251 GBM cells, sorted HOG‐A cells differing in TMZ resistance did not differ in cell size (Figure 6d,f).

HOG‐A cells sorted with the HOAPES‐3 device showed a gradation of membrane capacitance values across the sorted fractions, with cells in outlet 1 having the highest membrane capacitance values (Figure 6g). Cells in outlet 1 had the lowest midpoint membrane frequency, while there was no difference in membrane conductance or midpoint cytoplasm frequency of cells across the outlets (Figure 6h–j). Cells with the highest TMZ resistance were enriched in outlet 1, and there was no difference in cell size among the sorted cells (Figure 6k,l). These data show TMZ‐resistant cells can be enriched from multiple glioma types using DEP and sorted cells do not differ in cell size.

Glioma cells are highly dynamic, so we tested whether electrophysiological properties and TMZ resistance of enriched cells are maintained after sorting. D54 cells sorted in the HOAPES‐3 device showed the same pattern for membrane capacitance and midpoint membrane frequency immediately after sorting and 3 passages later, which is roughly 12 days post‐sort (Figure S9a,b). Similarly, membrane capacitance of sorted HOG‐A cells showed the same pattern immediately after sorting and 2–3 passages later, which corresponds to 15–20 days post‐sort (Figure S9c). The pattern of TMZ resistance of sorted cells showed the same pattern at 1 and 2 passages after HOG‐A cell sorting (Figure S9d) and was consistent through 4 passages (~25 days post‐sort) after HOG‐A cell sorting (Figure S9e). These results show that glioma cells can be sorted on the basis of TMZ resistance and that enrichment of TMZ‐resistant cells is maintained over several passages post‐sorting, thus providing an extended time frame for downstream analyses of sorted cells.

### 2.6 Separation of patient‐derived GBM TMZ‐resistant cells by electrophysiological properties

A rapid, label‐free means for enriching cells from patient tumors based on TMZ resistance could open new avenues of discovery and patient treatment options. As proof‐of‐concept, we tested whether DEP‐based separation of GBM cells recently derived from patient tumors would yield TMZ‐resistant cells. We optimized TMZ assays for DB93 patient‐derived GBM cells and found 7 days of TMZ incubation gave the most reliable IC_50_ values (Figure S10a). Analysis of cell viability revealed lower viability of DB93 GBM cells in DEP buffer, and viability was not improved by the addition of ROCKi (Figure S10b). We therefore tested DEP buffer supplemented with CEPT, a cocktail of factors shown to improve the viability of multiple cell types in culture.^33^ Addition of the CEPT cocktail improved the viability of DB93 GBM cells in DEP buffer (Figure S10b) without altering TMZ resistance (Figure S10c) and was used for DEP‐based analysis and sorting of DB93 GBM cells.

**FIGURE 7:**
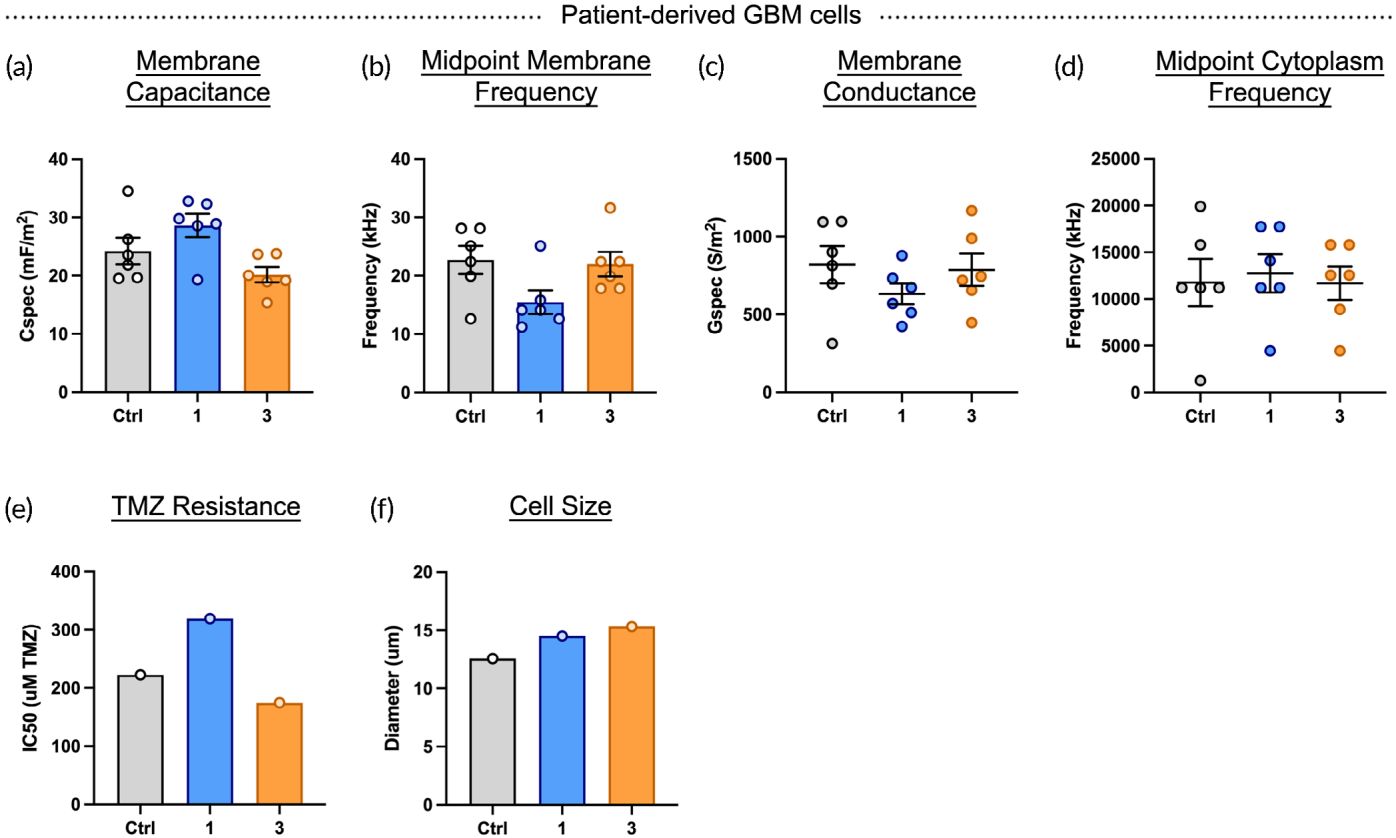
TMZ‐resistant cells isolated from patient‐derived GBM cells. (a) DB93 GBM cells from recently resected patient tumors were sorted into focused (outlet 1) and unfocused (outlet 3) fractions. Cells in outlet 1 had higher membrane capacitance values than cells in outlet 3 or controls (cells in DEP buffer and exposed to electric fields). (b) DB93 GBM cells in the unfocused outlet 3 had higher midpoint membrane frequency values compared to cells in the focused outlet 1 fraction. (c) Membrane conductance was slightly higher for cells in outlet 3 compared to those in outlet 1. (d) No clear difference was evident in midpoint cytoplasm frequency across the samples. (e) DB93 GBM cells in outlet 1 were more resistant to TMZ than those in outlet 3 or control. (f) Sorted cells were slightly larger in diameter than unsorted cells. Error bars show SEM for technical replicates, *n* = 1.

DB93 GBM cells were sorted in the HOAPES‐3 device, and analysis focused on cells collected in outlets 1 and 3 since they differed the most in membrane capacitance (Figure S10d). Sorted DB93 GBM cells differed in membrane capacitance and midpoint membrane frequency, and cells with the highest capacitance and lowest midpoint membrane frequency were those isolated in outlet 1 (Figure 7a,b). Of note, none of the DB93 GBM cell controls for sorting differed in membrane capacitance or midpoint membrane frequency (Figures S10e,f). There were slight differences in membrane conductance of cells in outlets 1 and 3, but no clear pattern emerged when comparing the results to those of other sorted glioma cells (Figures 7c, 6c,i, 5c). Midpoint cytoplasm frequency did not differ across the sorted DB93 GBM cells (Figure 7d). Analysis of TMZ resistance showed DB93 GBM cells with the highest resistance were collected in outlet 1, showing enrichment of resistant cells from recently resected patient tumor GBM cells (Figure 7e). Interestingly, the pattern of enrichment is similar to that observed for HOG‐A cells, which could relate to the fact that both patient‐derived DB93 GBM and HOG‐A cells were cultured in serum‐free conditions (Figures 6d,k, 7e). Sorted DB93 GBM cells were slightly larger in diameter than controls, which were not seen with other glioma cells, but the size of cells sorted to outlets 1 and 3 was similar (Figures 7f, 6f,l, 5f).

## 3 DISCUSSION

Our findings demonstrate that (a) TMZ‐resistant glioma cells can be separated without labels and solely on the basis of cell intrinsic properties, and (b) TMZ resistance is correlated with whole‐cell membrane capacitance and cell surface glycosylation. These findings could lead to rapid separation methods for patient tumor cells, enabling testing of alternative chemotherapeutics for personalized treatment and detailed molecular characterization of patient samples that could identify common resistance mechanisms.

Sorting to enrich TMZ‐resistant cells has several advantages over long‐term growth in TMZ to select resistant clones, which can induce cell characteristics not typically seen in TMZ‐resistant cells in patient tumors. Continuous cell growth in TMZ does not accurately mimic the development of resistant cells in patients since TMZ exposure in culture is greatly prolonged compared to the timing of TMZ treatment for patients.^25^ Additionally, cells cultured in TMZ may not recapitulate phenotypes of cells inherently resistant to TMZ that exist within the tumor prior to TMZ treatment. Our data show D54‐TR and U251‐TR cells selected by TMZ culture were larger in diameter than controls not grown in TMZ, which could lead to the interpretation that acquired resistance increases cell size. However, there was no association between cell size and TMZ resistance in sorted cells, suggesting cell size is not a good indicator of resistance. Another advantage to sorting is speed; TMZ‐resistant cells can be rapidly enriched by sorting whereas the process of deriving TMZ‐resistant cells in culture can take months. Utilizing cell intrinsic properties to enrich clinically important TMZ‐resistant cells from tumors is a novel method that can provide a realistically timed pipeline for molecular characterization and testing of alternative therapeutics for more tailored and effective treatment options to improve patient outcomes.

For sorting TMZ‐resistant cells, we used two types of DEP‐based microfluidic sorting devices. The HOAPES device^22^ sorts cells into two fractions, while the modified HOAPES‐3 generates three sorted cell fractions. Thus, an advantage of the HOAPES‐3 device is the ability to sort cells into fractions with a greater range of electrophysiological properties or cell sizes. For cells not differing in size, sorting into three fractions also enables greater separation of cells with the highest (outlet 1) and lowest (outlet 3) membrane capacitance values since mid‐range cells are directed to outlet 2. This can help to increase separation fidelity and improve downstream analysis of cell phenotype and gene expression patterns. We expected that cells sorted to outlet 2 would have intermediate TMZ resistance compared to cells in outlets 1 and 3, but this was not always the case (Figures 5, 6, 7). This could be due to variation in the percentage of cells directed to outlet 2 in each experiment or might indicate biological differences in cells with intermediate membrane capacitance that should be explored further. In the future, it will be important to continue optimizing sorting parameters and increasing the number of device outlets to fine‐tune the enrichment of highly TMZ‐resistant glioma cells. Furthermore, new technologies combining machine learning algorithms with cell sorting could increase sorting fidelity.^34^ For example, a supervised learning approach was successfully used with impedance cytometry to distinguish pancreatic cancer cells and cancer‐associated fibroblasts.^35^ Impedance cytometry, like DEP, detects cell‐intrinsic electrophysiological properties.^36^ Incorporating a similar approach with the HOAPES‐3 device in which individual cell electrophysiological properties are characterized and machine learning algorithms are implemented to tailor sorting parameters could improve specific cell targeting to the different outlets.

Cell intrinsic properties detected by DEP have been used extensively to identify and enrich cancer cells, but few studies have focused on glioma and chemotherapeutic resistant cells.^17^, ^37^, ^38^, ^39^ U251 GBM cultures enriched in cancer stem cells through growth as non‐adherent spheres differed from monolayer cells in their responses to DEP.^40^ These data may be consistent with TMZ‐resistant cell detection by DEP since cancer stem cells tend to be more chemotherapeutic resistant; however, cell electrophysiological properties and TMZ resistance were not directly measured in the study. In a separate study, membrane capacitance of five glioma cell lines correlated with p53 and PTEN expression.^41^ For other cancer cell types, impedance measurements identified prostate and breast cancer cell phenotypes and responses to drug treatment.^39^, ^42^, ^43^ Many of these studies utilized impedance measurements of cell monolayers, which reflect individual cell properties as well as cell–cell and cell–ECM adhesions. In a model of highly drug‐resistant circulating tumor cells, live pancreatic ductal adenocarcinoma cells were separated from apoptotic cells using microfluidic devices coupling DEP and deterministic lateral displacement (DLD) modules.^44^ Thus, DEP‐based sorting can be applied to many different cancer types to enrich chemotherapeutic resistant cells.

Earlier studies suggest cancer cell chemotherapeutic resistance relates to cytoplasmic conductivity. MCF7 breast cancer cells were cultured in chemotherapeutics to generate cells resistant to either paclitaxel or doxorubicin. The resistant cells differed from parent MCF7 cells in cytoplasmic conductivity, but the pattern was dependent on the type of chemotherapeutic; paclitaxel‐resistant cells had lower cytoplasmic conductivity values than parent MCF7 cells, while doxorubicin‐resistant cells had higher values.^45^ Results may be more consistent across cells resistant to the same chemotherapeutic since doxorubicin‐resistant K562 leukemia cells had higher cytoplasmic conductivity than parent K562 cells, matching the pattern observed with MCF7 doxorubicin‐resistant cells.^46^ The exact nature of the relationship between cytoplasmic conductivity and drug resistance is not clear since treating doxorubicin‐resistant K562 cells with a P‐glycoprotein drug efflux pump inhibitor reduced their doxorubicin resistance to the level of parent cells but did not change cytoplasmic conductivity.^46^ We did not observe differences in cytoplasmic conductivity across differentially TMZ‐resistant glioma cells, suggesting possible differences in resistance mechanisms across distinct cancer cell types.

We found significant differences in whole‐cell membrane capacitance and midpoint membrane frequency of DEP‐sorted glioma cells differing in TMZ resistance (Figures 5, 6, 7). These differences were also observed in TMZ‐resistant cells derived from extended culture in TMZ (Figure 2). Notably, membrane electrophysiological properties tracked with TMZ resistance and enabled enrichment of resistant cells from multiple glioma types. Sorting cells using electrophysiological properties may be effective for targeting chemotherapeutic resistant cells from other tumor types, since membrane capacitance distinguishes stem cell fate across multiple stem cell lineages and resistant cells often act as cancer stem cells, leading to new tumor growth.^15^ Thus, cell intrinsic properties provide robust measures of chemotherapeutic resistance in gliomas and must be explored further.

Membrane capacitance detected by DEP reflects plasma membrane composition and topography and is sensitive to cell surface glycosylation. We previously discovered that N‐linked glycosylation, particularly the N‐glycan branching pathway, regulates both membrane capacitance and fate bias of neural stem and progenitor cells.^9^, ^10^ Here, lectin binding identified differences in N‐glycans between control and TMZ‐resistant glioma cells. Compared to controls, TMZ‐resistant cells had lower binding of E‐PHA lectin, which detects bisecting N‐glycans, and DSL lectin, which binds β1–4 branched N‐glycans (Figure 1). The N‐glycan branching pathway is a key regulator of cancer cell function and controls a wide array of cell surface proteins that could contribute to drug resistance, including adhesion proteins, growth factor receptors, membrane transporters, and ion channels.^47^ However, the branching pathway's role in chemotherapeutic resistance has not been extensively studied. We also identified distinct patterns of fucosylation on control and TMZ‐resistant cells. TMZ‐resistant cells had lower levels of outer fucose (detected by AAL lectin) compared to controls (Figure 1). Fucose containing N‐glycans have been useful for identifying cancerous cells and determining prognosis, but more studies are needed to assess fucosylation's role in chemotherapeutic resistance.^48^ Taken together, N‐glycosylation is an important regulator of glioma cells and should be further studied.

Glycosylation can modulate cell stiffness by contributing to the formation of lipid rafts, which are cholesterol‐rich signaling centers, and by regulating the activity of cell surface adhesion molecules that link the extracellular matrix to the internal cytoskeleton.^47^, ^49^ Cell and matrix stiffness are altered in cancer, creating a complex interplay between tumor cell and microenvironmental mechanics.^50^ Microdevices that measure cell stiffness and deformability as well as impedance can distinguish pancreatic cancer cells from tumor‐associated fibroblasts, indicating the importance of assessing the physical characteristics of tumor cells.^51^ Glioma cells differing in glycosylation, membrane capacitance, and chemotherapeutic resistance may have additional biophysical properties, such as stiffness, that can be exploited for their enrichment from patient tumors.

In summary, glioma cells that differ in TMZ resistance can be sorted in a label‐free system based on their distinct plasma membrane characteristics. Cells that differ in TMZ resistance also vary in glycosylation, which regulates plasma membrane proteins tied to resistance mechanisms. It will be critical in the future to further assess plasma membrane characteristics of TMZ‐resistant cells from patient tumors.

## 4 METHODS

### 4.1 Ethics statement

Ethical approval for this study was obtained from the University of California, Irvine (UCI) Institutional Review Board (HS# 2012‐8912). DB93 cells were isolated from excess surgical tissue of resected patient tumors collected at UCI Medical Center following institutional guidelines, with previous written informed consent and strict observance of legal and institutional ethical regulations. This manuscript was prepared without the use of artificial intelligence large language models.

### 4.2 Glioma cell culture

D54‐MG (GBM, RRID: CVCL_5735), U251‐MG (GBM, RRID: CVCL_0021), and temozolomide (TMZ)‐resistant cells D54‐TR and U251‐TR were grown as adherent cultures in medium containing serum, while HOG‐A cells (RRID: CVCL_D354) were grown as non‐adherent spheres in serum‐free medium with FGF and EGF. For some experiments, HOG‐A cells were grown as adherent cultures in serum (HOG‐A‐S) for 3–6 weeks before analysis. DB93 (GBM) patient‐derived cells were grown as adherent cultures on laminin in serum‐free medium with FGF and EGF. Short tandem repeat testing (Labcorp) confirmed the identity of D54 (also known as A‐172), D54‐TR, U251, U251‐TR, HOG‐A cells, and DB93 patient cells did not match any cell lines. All cells were routinely screened for mycoplasma to ensure lack of contamination. Additional details in Appendix S1.

### 4.3 DEP analysis and buffers

Buffers for DEP experiments were as previously described for human and mouse cells.^22^, ^23^ RHO/ROCK pathway inhibitor (ROCKi) Y‐27632 (Stem Cell Technologies, 72304) was added to the DEP buffer at concentrations ranging from 1 to 10 μM. DEP buffer was supplemented with 1× CEPT cocktail (final concentrations in buffer: 50 nM chroman 1, MedChem Express, HY‐15392; 5 μM emricasan, SelleckChem, S7775; 1× polyamine supplement, Sigma‐Aldrich, P8483; 0.7 μM trans‐ISRIB, R&D Systems, 5284) instead of ROCKi for DB93 patient‐derived GBM cells. Cell responses to DEP electric fields (DEP spectra) and electrophysiological properties (membrane capacitance, membrane conductance, midpoint membrane frequency, midpoint cytoplasm frequency) were measured with a 3DEP analyzer (LabTech, East Sussex, UK).^26^ In some cases, measurements for midpoint cytoplasm frequency could not be accurately obtained because the applied frequency range did not produce DEP spectra that sufficiently decreased in magnitude in the higher frequency range to give reliable values for the midpoint frequency. As a result, midpoint cytoplasm frequency was not reported for some samples.

### 4.4 Flow cytometry, cell viability and TMZ resistance assays

Lectin binding was assessed by flow cytometry of fixed single cells using lectins described in Table S1. Cell viability was assessed immediately after incubating cells in DEP buffers by trypan blue staining and after 2 days of recovery by XTT assays (Biotium, NC0551071). To measure TMZ resistance, cells were treated with a TMZ concentration range, surviving cells were measured (XTT assay), and IC_50_ values calculated in GraphPad Prism (Version 9.1.2, RRID: SCR_002798) using nonlinear regression analysis, specifically the log(inhibitor) versus normalized response equation and variable slope model.

### 4.5 Modeling and simulations in MyDEP

D54 and D54‐TR measured properties (membrane capacitance and conductance, cytoplasm conductivity and permittivity) were used to simulate DEP spectra using a single‐shell model in MyDEP; values provided in Appendix S1.^52^

### 4.6 Glioma cell sorting with the hydrodynamic oblique angle parallel electrode sorter device

DEP‐based HOAPES device was used to sort cells as previously described with modifications detailed in Appendix S1.^22^, ^23^ In brief, glioma cells at a concentration of 2–4 × 10^6^ cells/mL were loaded into the device and sorted using flow rate 8–13 μL/min, voltage 3.5–7.5 V peak to peak, and frequency 50–500 kHz. Controls include cells plated immediately in growth media after dissociation (media control), cells incubated in DEP buffer with 5 μM ROCKi or 1× CEPT for the duration of the sort (DEP control), and cells exposed to DEP electric fields but not sorted (frequency control, cells combined from all outlets).

### 4.7 Statistical analysis

Comparison of two samples utilized two‐tailed unpaired Student's *t*‐tests; more than two samples were analyzed by one‐way ANOVA with either Tukey's *post hoc* correction for multiple samples or Dunnett's *post hoc* correction for comparison of multiple samples to a control. Statistical analysis used GraphPad Prism. Independent biological repeats are listed as “*n*” in figure legends. Some schematics were created using BioRender (RRID: SCR_018361).

For detailed methods, see Appendix S1.

## AUTHOR CONTRIBUTIONS

Conceptualization, A.Y.L.J., D.A.B., L.A.F.; Methodology, A.Y.L.J., A.R.Y., L.A.F.; Investigation, A.Y.L.J., A.R.Y., J.N.H., N.S.L, V.P.D, C.C.R., J.D.; Resources, C.R.D, K.D., D.A.B., L.A.F.; Writing—Original Draft, A.Y.L.J.; Writing—Review and Editing, V.P.D., L.A.F.; Funding Acquisition, D.A.B., L.A.F.; Supervision, L.A.F.

## CONFLICT OF INTEREST STATEMENT

L.A.F., A.Y.L.J., J.N.H., and C.C.R. hold a patent in dielectrophoresis.

## Supporting information


**Appendix S1:** Supplementary information.

**Movie S1:** D54 cell sorting in the HOAPES device. Cells were in DEP buffer supplemented with 5 μM ROCKi and sorted using DEP electrodes actuated at 7.5 Vp‐p and 500 kHz frequency with 10 μL/min flow rate. When the electric field is on, a subset of cells are focused to the center outlet. When field is off, the cells remain unfocused and exit the outer channels.

**Movie S2:** HOG‐A cell sorting in the HOAPES‐3 device. Cells were in DEP buffer supplemented with 5 μM ROCKi and sorted using DEP electrodes actuated at 5 Vp‐p with 8 μL/min flow rate. The applied frequency begins at 1000 kHz then decreases to 800, 600, 400, 200, 100, and 0 kHz. At 1000 kHz, most cells are directed to the center of the channel and exit outlet 1. As frequency decreases, more cells exit outlets 2 and 3 as fewer cells exit outlet 1. The percentage of cells exiting outlet 3 continues to increase as frequency drops, and when the electric field is off (frequency 0 kHz), most cells exit outlet 3.

## Data Availability

The data that support the findings of this study are available from the corresponding author upon reasonable request.
