# Supplementary material for: Glioma chemotherapeutic resistance is tied to membrane electrophysiological properties and glycosylation
Source: Bioeng Transl Med. 2025 Sep 22;11(1):e70069. doi: 10.1002/btm2.70069 (PMC12821226; doi:10.1002/btm2.70069)
Supplement: Supplementary file 1 — Appendix S1: Supplementary information. [file BTM2-11-e70069-s002.pdf]

Supporting Information

Glioma chemotherapeutic resistance is tied to membrane electrophysiological properties and glycosylation

Alan Y.L. Jiang[1,2], Andrew R. Yale[2,3], J. Nicole Hanamoto[2,4], Nicole S. Lav[2], Vi Phuong Dang[2,3], Clarissa C. Ro[2,4], Christopher R. Douglas[5], Kaijun Di[4], Jacob Deyell[4], Daniela A. Bota[2,4,5,6], Lisa A. Flanagan[1,2,3,4]

[1] Department of Biomedical Engineering, University of California Irvine, Irvine, CA 92697, USA

[2]Sue & Bill Gross Stem Cell Research Center, University of California Irvine, Irvine, CA 92697, USA

[3]Department of Anatomy & Neurobiology, University of California Irvine, Irvine, CA 92697, USA

[4]Department of Neurology, University of California Irvine, Irvine, CA 92697, USA

[5]Department of Pathology & Laboratory Medicine, University of California Irvine, Irvine, CA 92697, USA

[6]Chao Family Comprehensive Cancer Center, University of California Irvine, Orange, CA, 92868, USA

Corresponding author: Lisa Flanagan, lisa.flanagan@uci.edu

Supplementary Figures

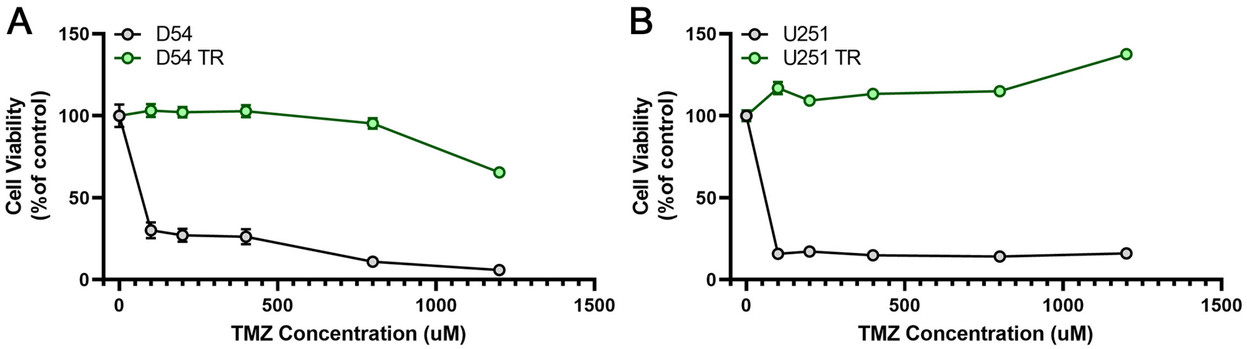

Figure S1: Control and TR GBM cells differ in resistance to temozolomide (TMZ). Control and TMZ-resistant (TR) D54 **(A)** and U251 **(B)** cells were exposed to increasing concentrations of TMZ and cell viability assessed after 7 days. TMZ dose response curves show that TR cells are more TMZ resistant than controls (n=1 each cell type, bars show SEM of technical replicates).

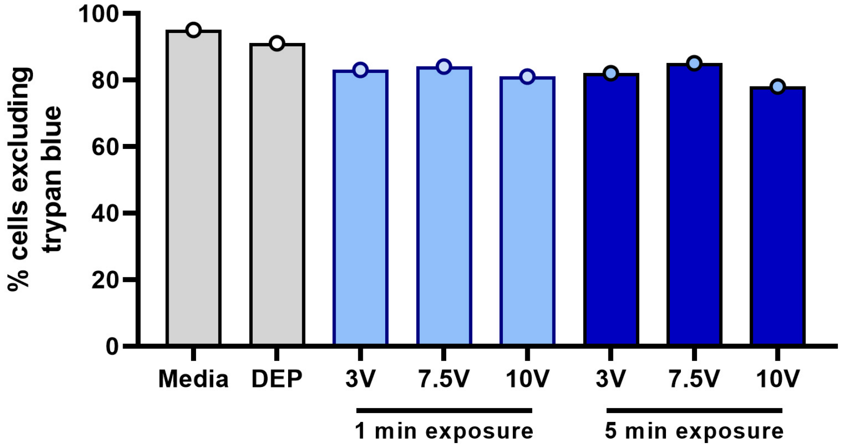

Figure S2: Electric field exposure is not toxic for D54 cells. D54 cells were exposed to DEP electric fields at 500 kHz and voltages ranging from 3-10 V for 1 or 5 minutes (~20-100 times longer than exposure in the DEP-based HOAPES device used for sorting). Electric field exposure did not significantly decrease cell viability (measured by trypan blue staining, n=1).

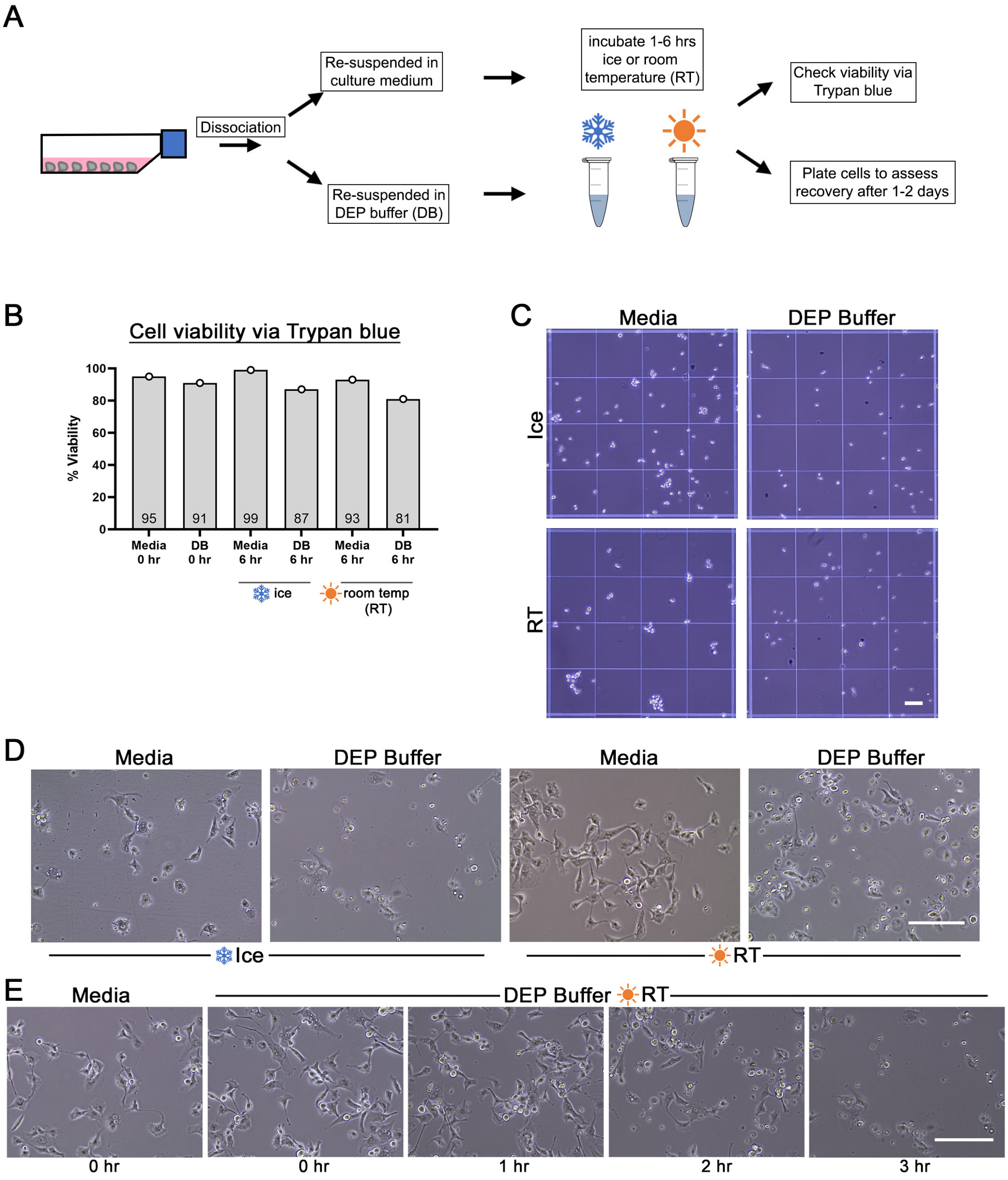

Figure S3: Incubation of D54 cells in DEP buffer can decrease viability. (A) Schematic of experimental paradigm to assess D54 viability after incubation in cell culture media or DEP buffer (DB). Adherent D54 cells were dissociated then resuspended in media or DEP buffer and incubated on ice or at room temperature (RT). Additional controls included cells resuspended in media or DEP buffer without incubation (0 hr). Cell viability was analyzed for each sample by trypan blue. Cell recovery was assessed by plating equal numbers of cells per condition and observing adherent cells after 1-2 days by phase-contrast microscopy. **(B)** Cell viability after 6 hour incubation remained high for all conditions. Viability of D54 cells in DEP buffer was only slightly lower than that of cells in media (n=1). **(C)** Phase-contrast images of D54 cells stained

with trypan blue after 6 hour incubation show high cell viability and increased clustering of cells in media compared to those in DEP buffer, particularly at RT. **(D)** Phase contrast images of adherent D54 cells one day after 6 hour incubation in media or DEP buffer. Cells incubated in media at RT show the best recovery, indicated by cell number and cell morphology. **(E)** Phase contrast images of adherent D54 cells one day after 0-3 hour incubation in media or DEP buffer at RT. Cell recovery decreases with increasing time of incubation in DEP buffer.

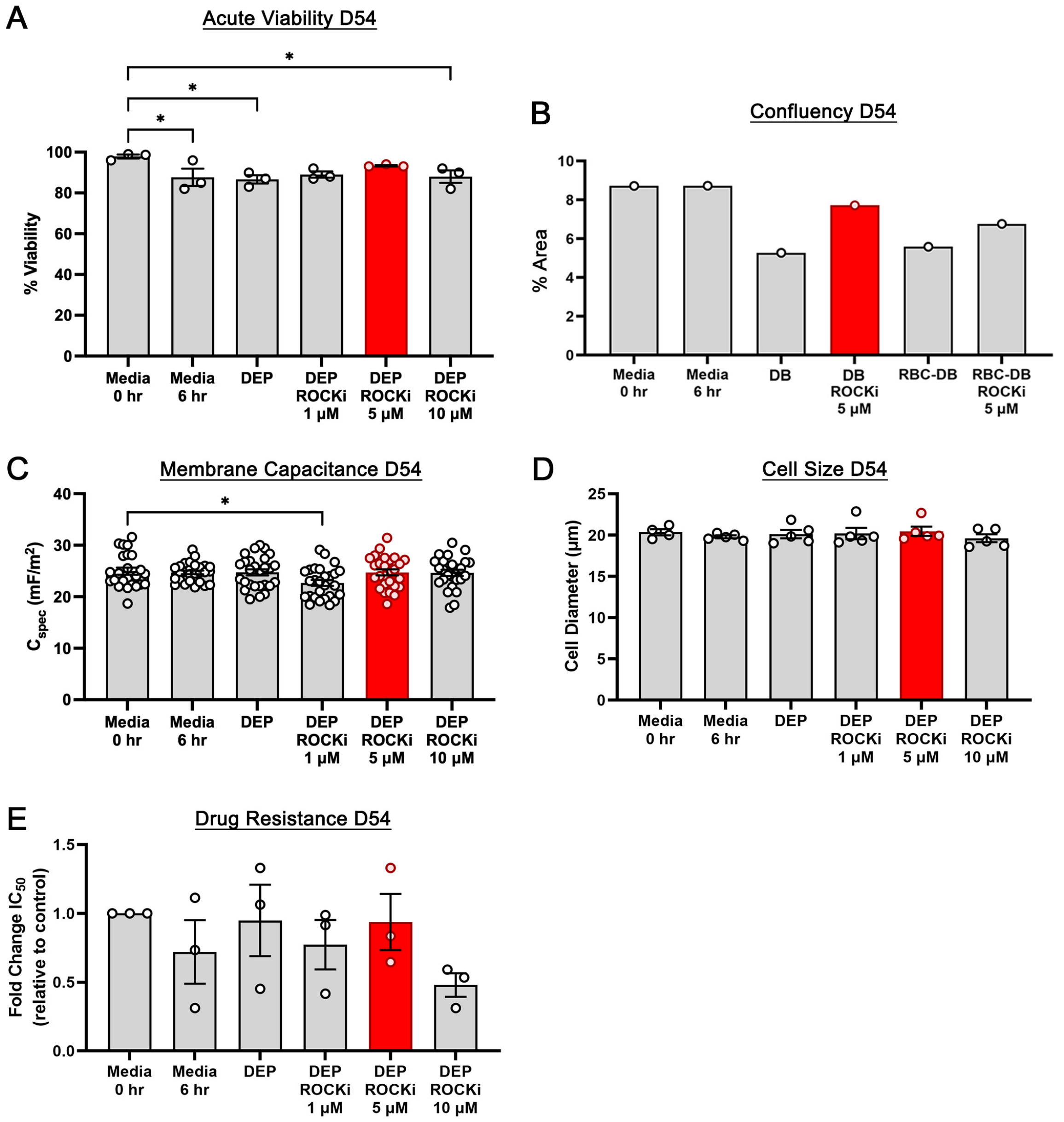

Figure S4: Optimization of ROCKi concentration in DEP buffer to support GBM cell viability. (A) Viability of D54 cells after 6 hour incubation in regular growth media, DEP buffer, or DEP buffer supplemented with 1, 5, or 10 μM ROCKi was measured by trypan blue staining. Acutely isolated cells (media control, 0 hr) served as a control. Viability of cells in DEP buffer with 5 μM ROCKi as not significantly different from that of control cells acutely isolated in media (Media, 0 hr). **(B)** D54 cells were incubated in media or buffers for 6 hours then plated. Confluency (% culture surface area covered by cells) of adherent cells was measured 1 day later using ImageJ. RBC-DB is RBC-DEP buffer (see Methods). Among cells incubated in DEP buffers (DB), those in DEP buffer supplemented with 5 μM ROCKi were the most confluent (n=1). **(C)** Incubation of D54 cells for 6 hours in DEP buffer with 5 μM or 10 μM ROCKi did not significantly impact membrane capacitance, although there was a slight difference for cells in buffer with 1 μM ROCKi. **(D)** There was no significant difference in the diameters of D54 cells in any of the conditions. **(E)** There was no significant difference in TMZ resistance as measured by fold change in IC_{50} relative to acutely isolated cells (media control, 0 hr). Error bars show SEM,

n≥3 (unless otherwise noted), all analyses one-way ANOVA, Dunnett's *post hoc* for multiple comparisons to control (Media, 0 hr).

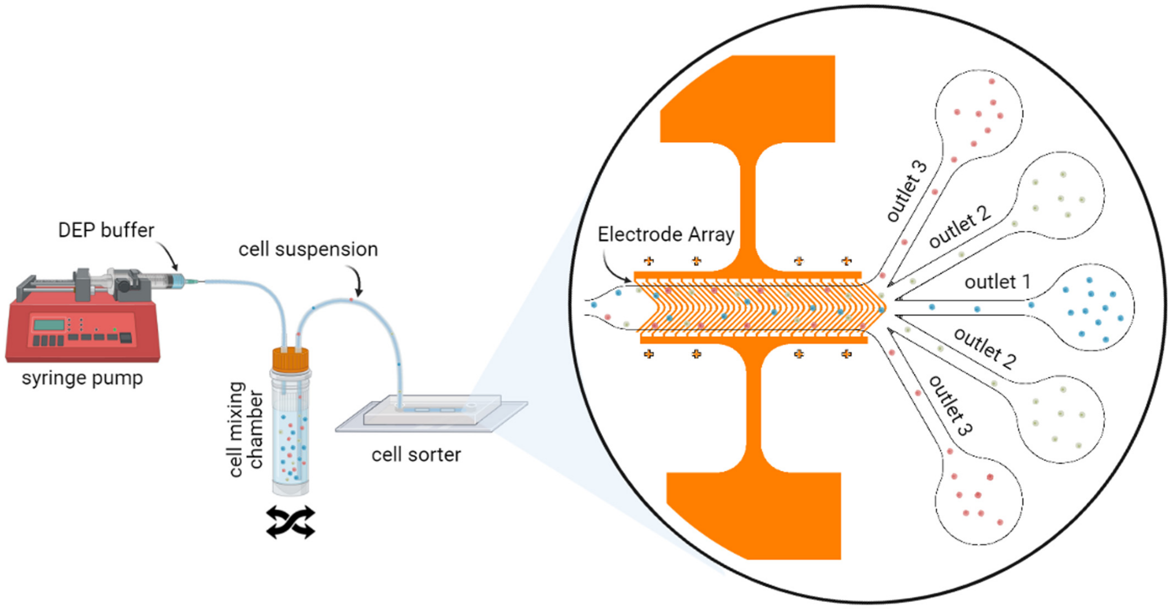

Figure S5: HOAPES-3 device with cell mixing chamber. A custom-designed cell mixing chamber was implemented to prevent cell settling prior to loading in the HOAPES-3 sorting device. A syringe pump was used to push DEP buffer into the cell mixing chamber pre-loaded with cells suspended in DEP buffer. The crossed arrows indicate intermittent mixing of the cell chamber via inversion to prevent cell settling. An Arduino-controlled motor (not shown) mixed the sealed tube containing cells every 15 seconds. The increased pressure in the chamber from the syringe pump causes the suspended cells to exit via tubing at the bottom of the chamber. Cells from the tubing then enter the device, go through the filter and hydrophoretic alignment sections, and enter the electrode array region along the edges of the channel. As the cells move through the electrode array region, the combined fluid force and induced DEP force cause cells experiencing positive DEP to be directed toward the center of the channel. Differences in the degree of positive DEP force cause cells to exit either outlets 1 or 2, and cells in negative DEP exit outlet 3.

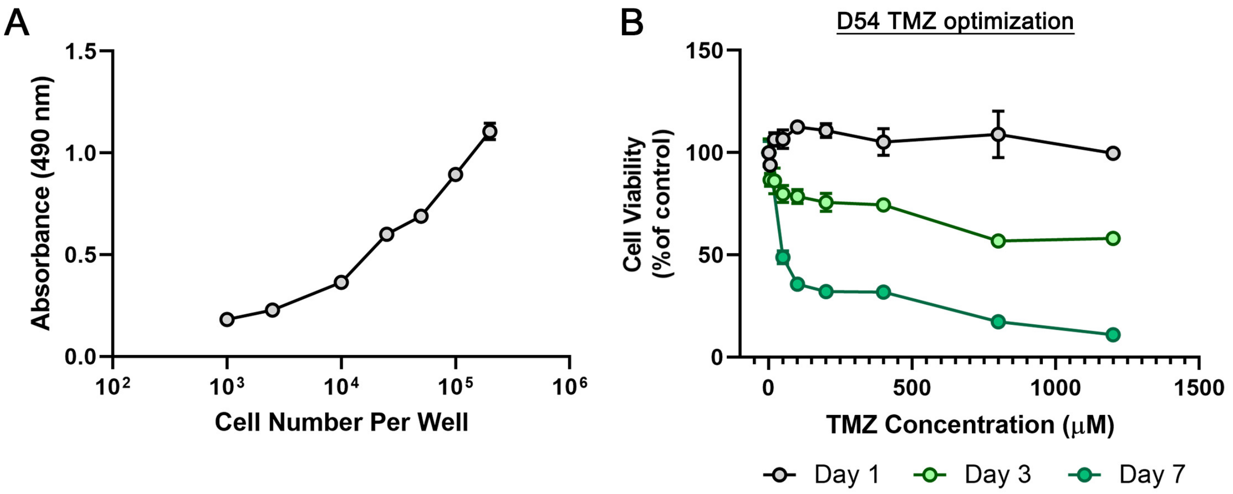

Figure S6: Optimization of TMZ assays for D54 GBM cells. (A) XTT assay shows increase in absorbance at 490 nm with increasing D54 cell density (number of cells per well). **(B)** D54 cells were grown in increasing concentrations of TMZ for 1, 3, or 7 days then viable cells were detected by XTT. TMZ treatment for 7 days is optimum to determine valid IC_{50} values since the percentage of viable cells drops below 50% and is quite low in the highest TMZ concentration (n=1). Error bars show SEM of technical replicates.

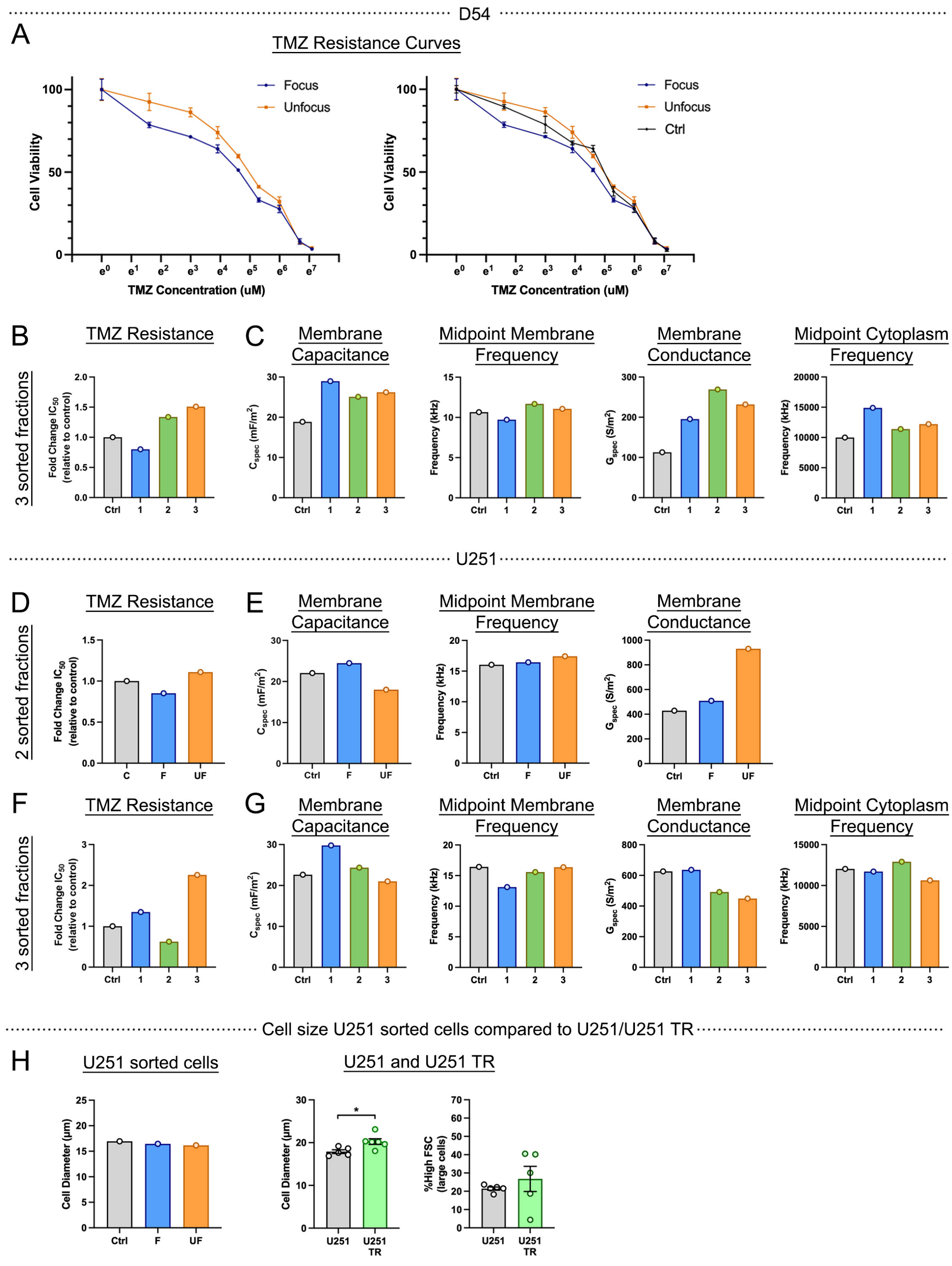

Figure S7: Sorted GBM cells differ in TMZ resistance and electrophysiological measures but not cell size. (A) Representative TMZ curves show differential TMZ sensitivities for cells in focused and unfocused fractions (left), with control cells shown relative to cells in focused and unfocused fractions (right) (axis is natural log). **(B)** D54 cells were sorted using the HOAPES-3 device and TMZ resistance was measured at 2 passages post-sorting. Fold change in TMZ

resistance relative to controls was lowest for D54 cells in outlet 1 and highest for cells in outlet 3 (n=1). **(C)** Membrane capacitance was measured for DEP sorted D54 cells and controls at 3 passages post-sorting. Cells with the highest membrane capacitance were in outlet 1. Graphs show midpoint membrane frequency, membrane conductance, and midpoint cytoplasm frequency of sorted cells and controls (n=1). **(D)** U251 cells were sorted using the HOAPES device and cells in the unfocused fraction were more resistant to TMZ than those in then focused fraction (n=1). **(E)** Membrane capacitance of sorted U251 cells was highest for cells in the focused fraction. Graphs show midpoint membrane frequency, membrane conductance, and midpoint cytoplasm frequency of sorted cells and controls (n=1). **(F)** The HOAPES-3 device was used to sort U251 cells and cells in outlet 3 had he highest fold increase in TMZ resistance relative to controls (n=1). **(G)** U251 cells sorted to outlet 1 had the highest membrane capacitance values. Graphs show midpoint membrane frequency, membrane conductance, and midpoint cytoplasm frequency of sorted cells and controls (n=1). TMZ resistance and membrane capacitance graphs in D, E, F, and G are the same as shown in Figure 5 and are replicated here for comparison to graphs of midpoint membrane frequency, membrane conductance, and midpoint cytoplasm frequency. **(H)** U251 cells sorted in the HOAPES device did not differ in size, analyzed by phase contrast microscopy. Analysis of U251 and U251-TR cell phase contrast images and forward scatter profiles in flow cytometry show that TR cells are significantly larger than controls (error bars show SEM, n≥3, unpaired Student's t-test control vs. TR).

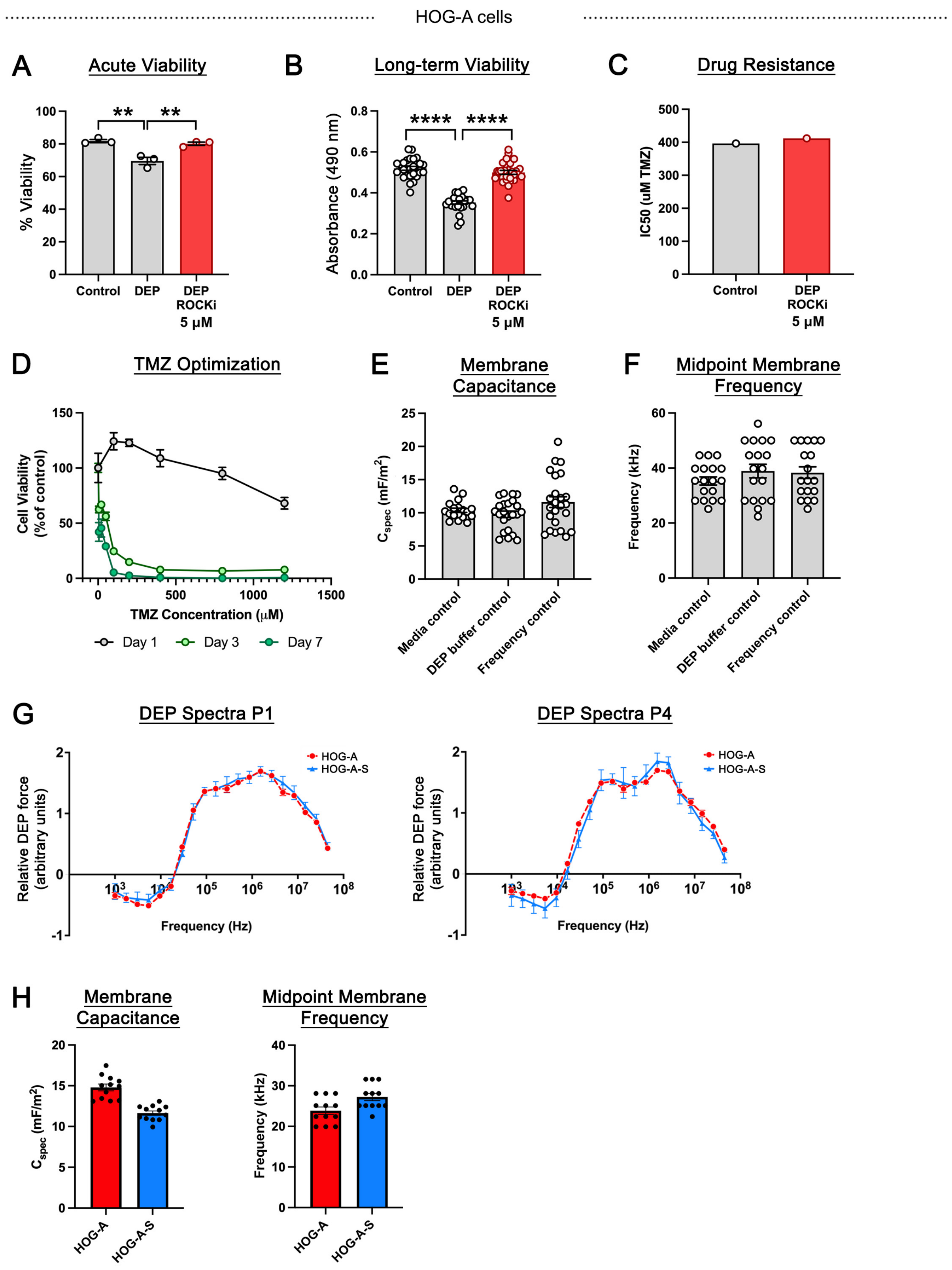

Figure S8: DEP sorting of HOG-A malignant astrocytoma cells. (A) Viability of HOG-A cells immediately after 6 hour room temperature incubation in regular growth media (control), DEP

buffer, or DEP buffer supplemented with 5 μM ROCKi was measured by trypan blue staining. Addition of ROCKi improved viability in the DEP buffer. **(B)** After 6 hour incubation in media or buffers, HOG-A cells were plated in normal growth conditions and the number of cells measured after 2 days by XTT assay. DEP buffer with 5 μM ROCKi significantly increased the number of viable cells compared to DEP buffer alone. **(C)** After 6 hour incubation in buffers, HOG-A cells were plated and allowed to recover for 1 day before treatment with TMZ to assess resistance. No difference in TMZ resistance was detected between media control and cells incubated in DEP buffer with 5 μM ROCKi (n=1). **(D)** HOG-A cells were cultured in increasing concentrations of TMZ for 1, 3, or 7 days and remaining viable cells measured by XTT. TMZ treatment for 3 days is optimum to determine valid IC_{50} values since the percentage of viable cells drops below 50% and is quite low in the highest TMZ concentration tested (n=1). Error bars show SEM of technical replicates. **(E)** HOG-A cell controls for DEP sorting included media control, cells incubated in DEP buffer throughout the duration of sorting (DEP buffer control), and cells in DEP buffer that were loaded into the device and exposed to electric fields but not sorted (frequency control). Controls did not differ in membrane capacitance (n≥3). **(F)** HOG-A cell controls for DEP sorting did not differ in midpoint membrane frequency (n≥3). **(G)** HOG-A cells were grown in regular or serum-containing (HOG-A-S) media for one (P1) or four (P4) passages and DEP spectra were collected. Shifts in the cells' frequency responses are evident in the spectra after 4 passages in serum containing media (n=2 at each passage). **(H)** HOG-A membrane capacitance and midpoint membrane frequency values were analyzed after 4 passages in regular or serum-containing media (HOG-A-S). Growth in serum induced a decrease in membrane capacitance and increase in midpoint membrane frequency (n=2). Error bars show SEM, n=3 (unless otherwise indicated), one-way ANOVA, Tukey *post hoc* for multiple comparisons, **$p<0.01$, ****$p<0.0001$.

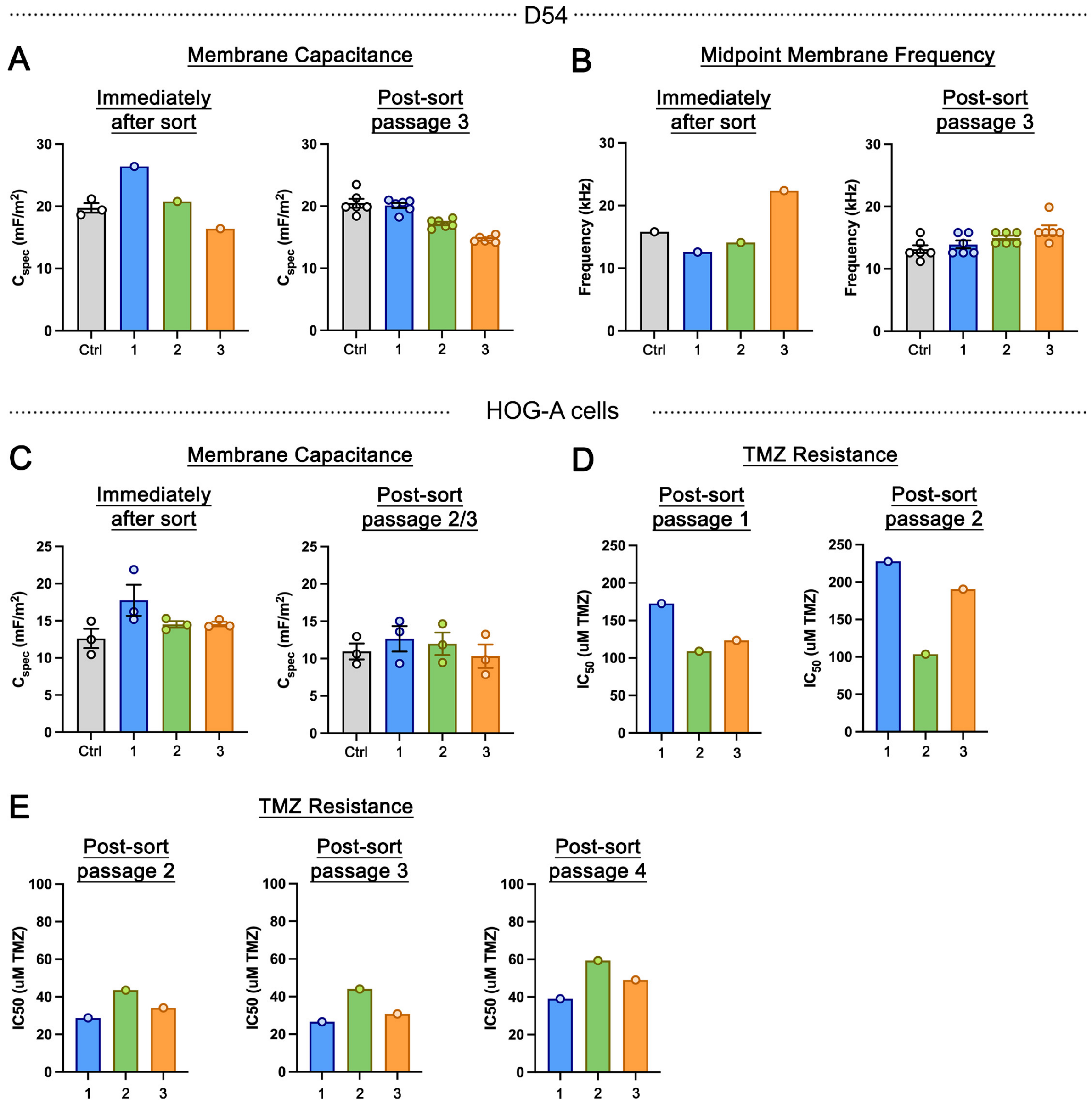

Figure S9: Differences in sorted cells' membrane capacitance and TMZ resistance are maintained after passaging. (A) D54 cells were sorted and membrane capacitance measured immediately after sorting and at 3 passages post-sorting (approximately 12 days after sorting). In both cases, cells with the highest membrane capacitance are in outlet 1, middle in outlet 2, and lowest in outlet 3 (n=1, same samples analyzed over time, fewer technical replicates for samples immediately after sorting due to limited numbers of cells). **(B)** Sorted D54 cells in outlet 3 have the highest midpoint membrane frequency, outlet 2 middle, and outlet 1 lowest. Sorted cells maintain that pattern after passaging. Same cells as shown in A. **(C)** HOG-A cells were sorted and membrane capacitance analyzed immediately after sorting. Cells with the highest membrane capacitance were in outlet 1 and those with lowest capacitance in outlet 3. A separate set of cells was analyzed at 2-3 passages after sorting (approximately 15-20 days post-sort), and showed the same membrane capacitance pattern (n=3). **(D)** Sorted HOG-A cells were analyzed for TMZ resistance at sequential passages, approximately 10 days and 15 days after sorting. Both show the same pattern of highest TMZ resistance for cells in outlet 1 and

lowest for outlet 2 (n=1, same samples analyzed over time). **(E)** Sorted HOG-A cells were analyzed for TMZ resistance at sequential passages and maintained the same pattern for approximately 25 days post sorting (post-sort passage 4, n=1, same samples analyzed over time). By about 30 days after sorting the pattern begins to degrade. Error bars show SEM of technical replicates.

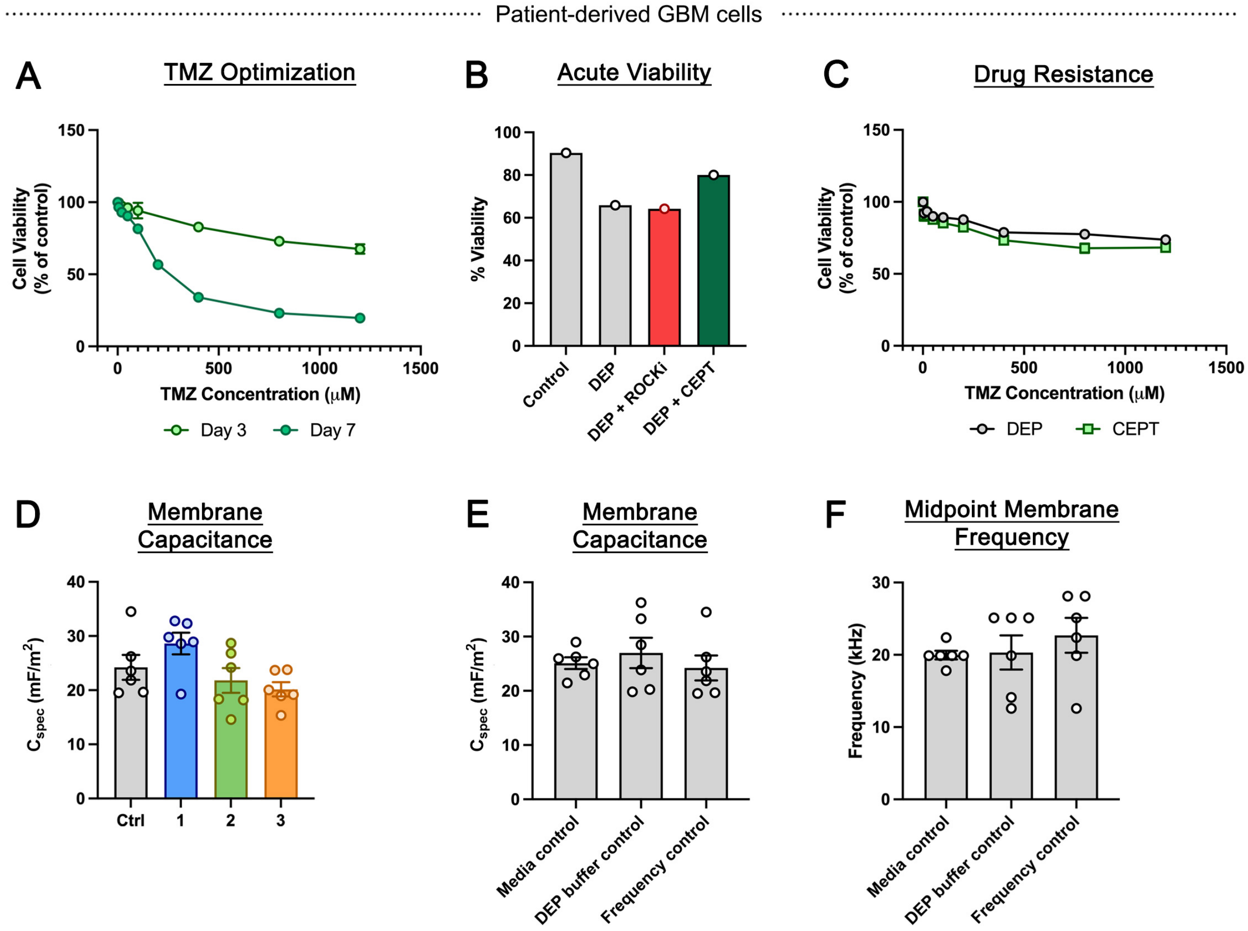

Figure S10: Controls for DEP sorting of patient-derived GBM cells. (A) DB93 patient-derived GBM cells were cultured in increasing concentrations of TMZ for 3 or 7 days and remaining viable cells measured by XTT. TMZ treatment for 7 days is optimum to determine valid IC_{50} values since the percentage of viable cells drops below 50%. **(B)** Viability of DB93 GBM cells immediately after 6 hour room temperature incubation in regular growth media (control), DEP buffer, DEP buffer supplemented with 5 μM ROCKi, and DEP buffer supplemented with CEPT was measured by trypan blue staining. Addition of CEPT improved DB93 viability in the DEP buffer. **(C)** After 6 hour incubation in buffers, DB93 GBM cells were plated and allowed to recover for 1 passage before treatment with TMZ to assess resistance. No difference in TMZ resistance was detected between cells in DEP buffer and those in DEP buffer with CEPT. **(D)** DB93 GBM cells from recently resected patient tumors were sorted into outlet 1, 2, and 3 fractions. Cells in outlet 1 had highest membrane capacitance values and those in outlet 3 had lowest. Control is cells in DEP buffer and exposed to electric fields (frequency control). **(E)** DB93 GBM cell controls for DEP sorting included media control, cells incubated in DEP buffer throughout the duration of sorting (DEP buffer control), and cells in DEP buffer that were loaded into the device and exposed to electric fields but not sorted (frequency control). Controls did not differ in membrane capacitance. **(F)** DB93 GBM cell controls for DEP sorting did not differ in midpoint membrane frequency. Error bars show SEM for technical replicates, n=1.

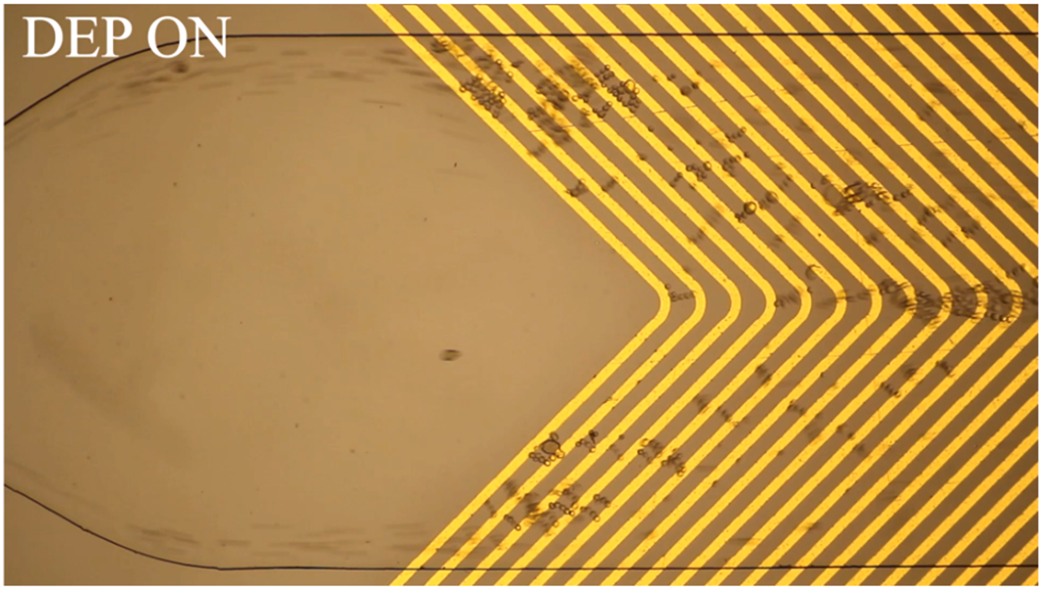

Sorting parameters: 500 kHz, 7.5 V, 10 uL/min

Movie S1: D54 cell sorting in the HOAPES device. Cells were in DEP buffer supplemented with 5 μM ROCKi and sorted using DEP electrodes actuated at 7.5 Vp-p and 500 kHz frequency with 10 ul/min flow rate. When the electric field is on, a subset of cells are focused to the center outlet. When field is off, the cells remain unfocused and exit the outer channels.

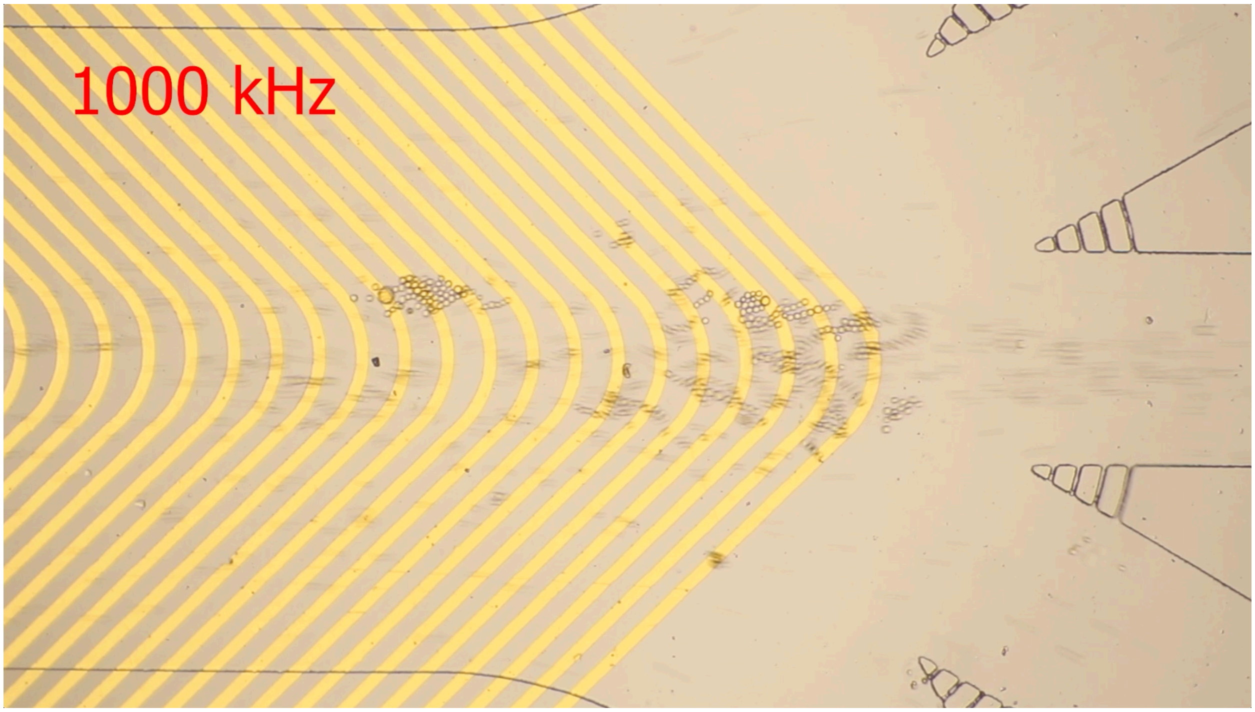

Sorting parameters: 1000-0 kHz, 5 V, 8 uL/min, 2X playback speed

Movie S2: HOG-A cell sorting in the HOAPES-3 device. Cells were in DEP buffer supplemented with 5 µM ROCKi and sorted using DEP electrodes actuated at 5 Vp-p with 8 ul/min flow rate. The applied frequency begins at 1000 kHz then decreases to 800, 600, 400, 200, 100, and 0 kHz. At 1000 kHz, most cells are directed to the center of the channel and exit outlet 1. As frequency decreases, more cells exit outlets 2 and 3 as fewer cells exit outlet 1. The percentage of cells exiting outlet 3 continues to increase as frequency drops, and when the electric field is off (frequency 0 kHz), most cells exit outlet 3.

Supplementary Methods

Glioma Cell Culture

Glioma cells in the study were D54-MG (GBM, RRID: CVCL_5735), U251-MG (GBM, RRID: CVCL_0021), DB93 patient-derived (GBM), HOG-A (oligodendroglioma/malignant astrocytoma, RRID: CVCL_D354), and temozolomide (TMZ)-resistant cells D54-TR and U251-TR; all cells were obtained from Dr. Daniela Bota's lab at University of California, Irvine (UCI). DB93 cells were originally isolated from excess surgical tissue of resected patient tumors collected at University of California Irvine (UCI) Medical Center following institutional guidelines, with previous patient consent and strict observance of legal and institutional ethical regulations. Protocols were approved by the Institutional Review Board (HS# 2012-8912) at UCI. GBM diagnosis was confirmed by a neuropathologist. TMZ-resistant cells were generated from D54 and U251 [1] by initially culturing the cells in 10 µM TMZ then increasing the TMZ concentration by 5-10 µM every three passages. The resulting resistant cell lines were maintained by culturing in 800 µM TMZ (Sigma Aldrich, T2577) for D54-TR and 600 µM TMZ for U251-TR every third passage. D54, D54-TR, U251, and U251-TR cells were grown in TC-treated T-25 flasks (Fisher Scientific, 10-126-28) in 50:50 DMEM and Ham's F12 medium (Corning, MT10092CV) containing 10% fetal bovine serum (FBS, Corning, MT35011CV) and 1% penicillin/streptomycin (pen-strep, Gibco, 15-14-122) in a humidified 37°C incubator with 5% CO2. Cells were passaged 1:3 at 90% confluency and were dissociated using non-enzymatic Cell Dissociation Buffer (Gibco, 130-15-016). DB93 patient-derived cells were cultured as adherent monolayers on laminin extracellular matrix coating (20 µg/ml, Thermo Fisher, 23017015) in Neurobasal Medium (Fisher Scientific, 10-888-022) containing 1X B27 (Gibco, 17504044), 20 ng/mL human FGF-basic (PeproTech, 100-18B), 20 ng/mL human EGF (PeproTech, AF-100-15), 1 mM sodium pyruvate (Gibco, 11360070), 2 mM L-glutamine (Fisher Scientific, 25030-081), and 1% pen-strep in a humidified 37°C incubator with 5% CO2. HOG-A cells were cultured as non-adherent spheres in the same supplemented serum-free media as DB93 cells. Half volume of media was replenished every 2-3 days. Spheres were dissociated for passaging by gentle mechanical trituration with a pipette. For some experiments, HOG-A cells were grown as adherent cultures in serum and thus denoted as HOG-A-S. HOG-A-S were cultured on TC-treated plates in 50:50 DMEM and Ham's F12 medium containing 10% FBS and 1% pen-strep in a humidified 37° C incubator with 5% CO2. Cells were passaged every 7 days or when confluent using Cell Dissociation Buffer. HOG-A-S were maintained in these conditions for 3-6 weeks before analysis. Cell authentication by short tandem repeat testing (Labcorp) confirmed identity of D54 (RRID: CVCL_5735, also known as A-172), D54-TR, U251 (RRID: CVCL_0021), U251-TR, and HOG (RRID: CVCL_D354) cells, and DB93 patient cells did not match any cell lines. All cells were routinely screened for mycoplasma to ensure lack of contamination (tested negative).

Buffers for DEP analysis

For DEP experiments, cells were dissociated into a single cell suspension using either (a) non-enzymatic Cell Dissociation Buffer for D54, D54-TR, U251, U251-TR and adherent HOG-A-S cells, (b) gentle mechanical trituration with a pipette for HOG-A cells, or (c) Accutase Cell Detachment Solution (Stem Cell Technologies, 07920) for DB93 patient-derived cells. Dissociated cells were washed 2-3 times with and resuspended in an iso-osmotic DEP buffer consisting of 8.5% (w/v) sucrose, 0.3% (w/v) glucose, and 0.7% (w/v) RPMI-1640 (Fisher Scientific, SH3025501) to adjust final conductivity to 100 µS/cm, measured with a conductivity meter (Thermo Orion, Beverly, MA). An alternative RBC-DEP buffer solution consisting of 250 mM sucrose, 17 mM glucose, 0.1 mM $CaCl_2$, 0.25 mM $MgCl_2$, with final conductivity adjusted to 100 µS/cm using 0.8% PBS [2] was also tested. RHO/ROCK pathway inhibitor (ROCKi) Y-27632 (Stem Cell Technologies, 72304) was added to the DEP buffer at concentrations ranging from 1-10 µM and cell viability, membrane capacitance, cell size, and TMZ resistance assessed as described below. DEP buffer was supplemented with 1X CEPT cocktail (final

concentrations in buffer: 50 nM chroman 1, MedChem Express, HY-15392; 5 μM emricasan, SelleckChem, S7775; 1x polyamine supplement, Sigma-Aldrich, P8483; 0.7 μM trans-ISRIB, R&D Systems, 5284) instead of ROCKi for DB93 patient-derived GBM cells.

DEP cell analysis

Cell responses to DEP electric fields and electrophysiological properties were measured with a 3DEP analyzer (LabTech, East Sussex, UK) [3-5]. Cells in DEP buffer were loaded into the 3DEP analysis chip at a concentration of 2 x 10^6 cells/mL and DEP spectra were generated by exposing cells to frequencies ranging from 1-45,000 kHz at 5 points per decade. Cell movement in response to the applied electric field was graphed as relative DEP force at each frequency (arbitrary units reflecting light intensity changes in the measurement wells that reflect relative DEP force) [3-5]. Electrophysiological properties (membrane capacitance, membrane conductance, midpoint membrane frequency, midpoint cytoplasm frequency) were calculated in the 3DEP analyzer software using the auto-fit single-shell spherical DEP polarization model. For some analyses, the applied frequency range did not extend high enough to reliably calculate midpoint cytoplasm frequency, in which case cytoplasmic values were not reported. Cell diameter was measured from phase contrast images of dissociated cells using an in-house ImageJ (RRID: SCR_003070) script. Cell diameters were included in the 3DEP analyzer software for calculation of electrophysiological properties.

Flow cytometry to assess lectin binding

To assess lectin binding, live cells were dissociated using non-enzymatic Cell Dissociation Buffer. Cells were fixed in 4% paraformaldehyde as dissociated single cells and re-suspended in PBS containing 5% BSA and 0.02% sodium azide. Cells were incubated with fluorescein-conjugated lectins for 1 hour on ice protected from light. See Table 1 for lectins and concentrations. After incubation, cells were centrifuged to remove excess staining solution, washed once with PBS, and re-suspended in PBS at a concentration of ~1 x 10^6 cells/ml. All cell samples were filtered through a 35 μm cell strainer (Falcon, 352235) to remove cell clumps. All cell samples were processed using a BD LSR Fortessa X-20 and BD FACSDiva software (RRID: SCR_001456). All data analysis was performed using FlowJo (RRID: SCR_008520) v10.1 or higher.

Table 1: Lectin reagents and concentrations

Reagent	Vendor	Catalogue	Final Concentration
Canavalia ensiformis concanavalin A (ConA), Fluorescein labeled	Vector Laboratories	FL-1001	20 μg/mL
Phaseolus vulgaris erythroagglutinin (E-PHA), Fluorescein labeled	Vector Laboratories	FL-1121	20 μg/mL
Datura stramonium lectin (DSL), Fluorescein labeled	Vector Laboratories	FL-1181	20 μg/mL
Phaseolus vulgaris leucoagglutinin (L-PHA), Fluorescein labeled	Vector Laboratories	FL-1111	20 μg/mL
Lycopersicon esculentum agglutinin (LEA, LEL), Fluorescein labeled	Vector Laboratories	FL-1171	50 μg/mL
Lens culinaris agglutinin (LCA), Fluorescein labeled	Vector Laboratories	FL-1071	20 μg/mL
Aleuria Aurantia Lectin (AAL), Fluorescein labeled	Vector Laboratories	FL-1391	20 μg/mL
Sambucus nigra agglutinin (SNA), Fluorescein labeled	Vector Laboratories	FL-1301	40 μg/mL

Cell viability and cell quantitation assays

Cell viability in response to DEP buffers was assessed by incubating cells in buffers for 1-6 hours at room temperature or on ice as noted for select experiments. Cell viability was quantified immediately after incubation using trypan blue staining. Cells were then plated in normal growth media to assess recovery after 1-2 days by analysis of phase contrast images of adherent cells and XTT (Biotium, NC0551071) assays to quantify viable cells. Phase contrast images were captured after 1-2 days of culture, and ImageJ was used to quantify the percentage of the total surface area covered by cells as a measure of confluency. For XTT assays, D54 and HOG-A cells incubated in DEP buffers were plated in triplicate at 2,500 and 5,000 cells/well in 50 µL of growth medium in a 96-well plate, respectively. Blank wells were plated as controls with equivalent volumes of media, all outer wells of the plate were filled with 200 µL PBS to reduce evaporation, and the plate was returned to the incubator for 2 days. Viable cells were assessed by adding 75 µL of activated XTT solution to each well and incubating for 4 hours at 37° C. Absorbance for each well was measured on an Azure c600 Molecular plate reader at 475, 490 and 660 nm. To test the linearity of the XTT assay, D54 cells were dissociated with Cell Dissociation Buffer and plated at a range of concentrations (1,000, 2,500, 10,000, 25,000, 50,000, 100,000, 200,000 cells/well). The cells were returned to the incubator and allowed to recover and adhere. After a 4 hour incubation, the XTT assay was performed and absorbance graphed as a function of cell concentration.

DEP electric field exposure

Viability of D54 cells in response to DEP electric fields was assessed in a device consisting of interdigitated electrodes at the bottom of a microwell [6]. Since there is no fluid flow in the well, shear stress on the cells is avoided to focus solely on effects of exposure to the electric fields. Cells were exposed to 500 kHz frequency at 3, 7.5, or 10 volts for 1 or 5 minutes. Cells were then removed from the well and trypan blue was used to calculate the percentage of viable cells.

Analysis of TMZ resistance

TMZ resistance of glioma cells was assessed by treating cells with a range of TMZ concentrations, measuring the number of surviving cells using an XTT assay, and calculating the TMZ IC_{50} value. Glioma cells were dissociated, resuspended in fresh culture media, and plated in triplicate at a density of 2,500 (D54), 5,000 (HOG-A), or 10,000 (DB93) cells/well in 50 µL of growth medium in a 96-well plate. Blank wells were plated with 50 µL of growth media. All outer wells of the plate were filled with 200 µL of PBS to reduce evaporation. Plated cells were allowed to recover for 24 hours before the addition of TMZ. A 200 mM TMZ stock solution (Sigma Aldrich, T2577) was prepared in DMSO then further diluted to various concentrations (0, 5, 20, 50, 100, 200, 400, 800, 1200 µM) with appropriate volumes of growth media and DMSO to ensure equal amount of DMSO for each condition. At the start of treatment, 50 µL of the adjusted TMZ solutions were added to wells for a total volume of 100 µL per well, and the cells were incubated for 3 (HOG-A) or 7 days (D54, D54-TR, U251, U251-TR, DB93; pilot experiments also tested 1- and 3-day incubations). Cell viability was assessed by XTT as described above. Values were normalized to DMSO controls and graphed as a function of TMZ concentration to generate TMZ curves. The IC_{50} values were calculated in GraphPad Prism (Version 9.1.2, RRID: SCR_002798) using nonlinear regression analysis, specifically the log(inhibitor) vs. normalized response equation and variable slope model.

Modeling and simulations in MyDEP

The measured electrophysiological properties of D54 and D54-TR cells (membrane capacitance and conductance, cytoplasm conductivity and permittivity) were used to simulate DEP spectra using MyDEP [7, 8]. Two hypothetical cells differing in membrane capacitance (10 and 20 mF/m^2) were modeled using a single-shell model. Other parameters were as follows: buffer

conductivity 100 µS/cm, buffer permittivity 80, frequency range 10^2-10^9 Hz at 100-point resolution, cell radius 10 µm for both D54 and D54-TR (measured mean radii were D54: 9.8 +/- 0.1 µm SEM and D54-TR: 10.8 +/- 0.3 µm SEM), cytoplasm conductivity 0.15 S/m, cytoplasm permittivity 60, and specific membrane conductance 400 S/m^2.

Glioma cell sorting with the hydrodynamic oblique angle parallel electrode sorter (HOAPES) device:
The HOAPES device is comprised of three main sections: filter, sheathless hydrophoretic cell aligner, and DEP separation module with oblique parallel electrodes. The design and fabrication of the HOAPES device were as previously described with the following modifications [9].

Two sorted fractions, three outlet HOAPES device: To better accommodate the size of glioma cells and improve diverging flow in the hydrophoretic cell aligner, the height of the multi-layered channel was increased from 40 µm to 54 µm for the second layer during fabrication. As cells encounter the non-uniform electric field in the DEP separation module, those with lower membrane capacitance experience negative DEP and remain at the channel walls to exit the outer channel outlets, while cells with higher capacitance experience a strong positive DEP force that focuses them to the center of the channel so they exit the middle outlet. Sorting yields two cell populations: focused in the middle outlet (higher capacitance) and unfocused in the outer outlets (lower capacitance).

Three sorted fractions, five outlet HOAPES-3 device: A new version of the HOAPES device (HOAPES-3) was created with additional outlets and double the number of electrode pairs to improve sorting resolution. The HOAPES-3 device has 5 outlets which results in 3 sorted groups (Outlet 1 from the single center channel, Outlet 2 from the 2 intermediate channels, and Outlet 3 from the 2 outer channels). During sorting, cells with low membrane capacitance remain along the channel walls to exit at Outlet 3, those with medium membrane capacitance get partially focused by positive DEP and exit at Outlet 2, and those with high membrane capacitance experience a strong positive DEP force that focuses them to the center of the channel to exit at Outlet 1. In addition to the increased number of outlets, the number of electrode pairs was expanded from 20 to 40, which increases DEP-induced separation when using the same sorting parameters.

For sorting in both HOAPES devices, cells were washed with and resuspended in DEP buffer with 5 µM ROCKi or CEPT for DB93 and filtered with 35 µm cell strainers to remove cell clumps. The final cell concentration was adjusted to 2-4 x 10^6 cells/mL and loaded into a custom-made cell mixing chamber that intermittently mixed the cell solution via inversion, which prolongs consistent cell delivery to the sorter by preventing cell settling. The cell mixing chamber utilized a Micro Servo motor (model MG996R) controlled by an Arduino Uno REV3 to actuate mixing of a sealed tube containing cells every 15 seconds. The cells were then flowed to the device from the cell mixing chamber through 0.5 mm inner diameter, 1.5 mm outer diameter Tygon tubing (VWR, Cat # 89404-302). The HOAPES device was mounted on an upright Olympus microscope (model BX41) with bright field objectives and connected to a function generator (AFG320, Tektronic, Beaverton, OR). A commercial dSLR camera (Canon model EOS Rebel T2i) was attached to the microscope to record videos and monitor sorting.

Sorting glioma cells in the HOAPES device was as previously described [9] and used the following parameters: flow rate 8-13 µL/min, voltage 5-7.5 V peak to peak, and frequency 500 kHz. The percentage of cells in the focused fraction averaged 42% +/- 15 s.d. for D54 cells, 40% for U251, 29% +/- 8 s.d. for HOG-A, and 56% +/- 4 s.d. for HOG-A-S. Sorting parameters for the HOAPES-3 device were: flow rate 1.5-8 µL/min, voltage 3.5 V, and frequency 50-100 kHz. The controls for DEP sorted cells include cells plated immediately in growth media after dissociation (Media Control, MC), cells incubated in DEP buffer with 5 µM ROCKi or CEPT for

the duration of the sort (DEP Control), and cells exposed to DEP electric fields but not sorted (Frequency Control, FREQ, cells combined from all outlets). Sorted cells were collected from each outlet of the device and either analyzed immediately or allowed to recover and undergo passaging before analysis.

Statistical Analysis:

Normally distributed data with comparison of two samples utilized two-tailed unpaired Student's t-tests. Normally distributed datasets containing more than two samples were analyzed by one-way ANOVA with either Tukey's *post hoc* correction for multiple samples or Dunnett's *post hoc* correction for comparison of multiple samples to a control. Datasets with missing values that were incompatible with ANOVA analysis were instead analyzed by mixed effects analysis and utilized Tukey's *post hoc* correction for multiple samples. Statistical analysis used GraphPad Prism. Independent biological repeats are listed as "n" in figure legends and were 3 or more in order to perform statistical analysis. Some schematics were created using BioRender (RRID: SCR_018361).

References

1. Bektas, M., S.P. Johnson, W.E. Poe, D.D. Bigner, and H.S. Friedman, A sphingosine kinase inhibitor induces cell death in temozolomide resistant glioblastoma cells. Cancer Chemother Pharmacol, 2009. **64**(5): p. 1053-8.
2. Fry, C.H., S.C. Salvage, A. Manazza, E. Dupont, F.H. Labeed, M.P. Hughes, and R.I. Jabr, Cytoplasm resistivity of mammalian atrial myocardium determined by dielectrophoresis and impedance methods. Biophys J, 2012. **103**(11): p. 2287-94.
3. Broche, L.M., F.H. Labeed, and M.P. Hughes, Extraction of dielectric properties of multiple populations from dielectrophoretic collection spectrum data. Phys Med Biol, 2005. **50**(10): p. 2267-74.
4. Hoettges, K.F., Y. Hubner, L.M. Broche, S.L. Ogin, G.E. Kass, and M.P. Hughes, Dielectrophoresis-activated multiwell plate for label-free high-throughput drug assessment. Anal Chem, 2008. **80**(6): p. 2063-8.
5. Broche, L.M., K.F. Hoettges, S.L. Ogin, G.E. Kass, and M.P. Hughes, Rapid, automated measurement of dielectrophoretic forces using DEP-activated microwells. Electrophoresis, 2011.
6. Lu, J., C.A. Barrios, A.R. Dickson, J.L. Nourse, A.P. Lee, and L.A. Flanagan, Advancing practical usage of microtechnology: a study of the functional consequences of dielectrophoresis on neural stem cells. Integr Biol (Camb), 2012. **4**(10): p. 1223-36.
7. Cottet, J., O. Fabregue, C. Berger, F. Buret, P. Renaud, and M. Frénéa-Robin. *MyDEP: a new computational tool for dielectric modeling of particles and cells*.
8. Cottet, J., O. Fabregue, C. Berger, F. Buret, P. Renaud, and M. Frenea-Robin, *MyDEP: A New Computational Tool for Dielectric Modeling of Particles and Cells.* Biophys J, 2019. **116**(1): p. 12-18.
9. Jiang, A.Y.L., A.R. Yale, M. Aghaamoo, D.H. Lee, A.P. Lee, T.N.G. Adams, and L.A. Flanagan, *High-throughput continuous dielectrophoretic separation of neural stem cells.* Biomicrofluidics, 2019. **13**(6): p. 064111.
